# Stepping up: A qualitative examination of youth Camogie players’ perceptions of playing up an age grade

**DOI:** 10.1371/journal.pone.0355563

**Published:** 2026-08-11

**Authors:** Sinéad O’Keeffe, Andrew Lane

**Affiliations:** 1 School of Health and Human Performance, Dublin City University, Dublin, Ireland; 2 Department of Health and Sport Sciences, South East Technological University, Carlow, Ireland; Technological University Dublin - Tallaght Campus, IRELAND

## Abstract

**Purpose:**

Playing up an age grade is a common phenomenon in youth Camogie, but there is no current understanding of the perception of youth players in doing so. This study aimed to utilise the athlete voice to qualitatively examine youth Camogie players’ perceptions of playing up an age grade within the sport.

**Methods:**

Semi-structured interviews were conducted with adolescent club Camogie players currently playing up an age grade (n = 15; 14.4 ± 1.1 years). Interviews were audio-recorded and the transcripts were analysed using reflexive thematic analysis.

**Results:**

Despite initial difficulties encountered, participants ultimately enjoyed playing up, reporting enhanced performance and skill development, alongside increased psychosocial competence. However, the pressures and demands of playing up, negative peer interactions, and impaired intrinsic motivation were acknowledged as perceived negatives, but strategies such as improved social inclusion, coach and older player support, and player autonomy were suggested to facilitate a better experience.

**Conclusion:**

The findings highlight the need to provide developmentally appropriate opportunities for youth Camogie players that support players’ progression, while also being cognisant of the risks of injury and burnout. Coaches play a critical role in enabling these opportunities, but player autonomy should ultimately guide decision-making. When supported effectively, positive playing up experiences can foster competence and confidence, contributing to improved player retention.

## Introduction

Camogie is an invasion-based Gaelic games team sport played by youth and adult female athletes [1]. Governed by the Camogie Association, Camogie is one of the most popular community and school participatory sports for primary and post-primary school girls [2]. Competitive youth Camogie participation typically takes place within formal structures from under-14 onwards (including under-15, under-16, under-17 and/or under-18 age grades). However, many players begin engaging with Camogie at much younger ages (e.g., under-6 or under-8), where early participation focuses on skill development, game-based learning, meaningful playing time and enjoyment of the sport [3]. Sports participation is widely championed due to its diverse developmental benefits for youth, spanning from physical improvements, such as muscular strength and endurance, to psychological and social gains, such as quality relationships and increased self-esteem [4]. Despite the well-documented benefits of participation, over 70% of youth sports participants in Ireland drop out of sport, with 15% dropping out of Camogie specifically, positioning it among the most commonly dropped sports young girls cease participating in [2].

In Camogie, a player is deemed eligible to play and compete at a certain grade based on age criteria determined by their chronological age (i.e., number of years of age, calculated by birth date). Playing up an age grade, considered playing a chronological age grade above the current primary age grade for that player, is common for youth Camogie players, with 52% of under-14 female Gaelic games players playing up to under-16 and 72% of under-16s playing up to under-18 [5]. Playing up is often permitted or encouraged when an individual is deemed more skilled than their same-aged peers [6] or when participation numbers are lacking and youth players are required to field teams [7]. Playing up is considerably more prevalent for young females in rural settings in Ireland, with 74% of under-14 players doing so, compared to 22.7% in towns and just 3.3% in cities [5]. Despite its prevalence, there is a current lack of research exploring the phenomenon, limiting the extent to which its potential benefits or associated developmental demands can be adequately evaluated.

Playing alongside players with a greater chronological, biological and technical age can be beneficial for youth players as more appropriate levels of challenge can lead to better performances and more skilled players [8]. The technical, tactical and/or physical benefits associated with playing up have been demonstrated to date in boys youth soccer [9] and rugby [10]. Additionally, there are psychological and social benefits of playing up, such as making new friends and developing social interaction skills [9], which can support adolescents’ social functioning, group cohesion and sport continuation behaviour [4,11]. Previous qualitative research in youth boys and girls has identified progress as an athlete as a key benefit of playing up in soccer, with receiving recognition for their talent, experiencing success and having opportunities to develop expertise highlighted [6]. However, these young athletes can find playing up a challenge and struggle most to cope with the intensity of trainings and games, and to fit in socially with older peers [6]. There are also associated risks of injury, burnout and overtraining [12], which must be avoided in order to reduce female dropout from sport and physical activity during adolescence.

There is no current research examining the effects of or the perceptions of youth players towards playing up an age grade in Camogie, or even in Gaelic games as a whole. Therefore, this study aimed to qualitatively examine youth Camogie players’ perceptions of playing up an age grade within the sport. In line with this aim, the objectives were to gain an understanding of why youth Camogie players currently play up an age grade, their perceived benefits and negatives of playing up, and their recommendations for future players considering playing up an age grade.

## Materials and methods

### Design

This study adopted a qualitative phenomenological approach, utilising semi-structured interviews to examine youth Camogie players perceptions of playing up an age grade. Phenomenology seeks to understand the essence of lived experiences by exploring individuals’ subjective perspectives, enabling a rich and in-depth understanding of what matters to participants and how they make sense of their experiences [13]. Qualitative research in this sense specifically aims to integrate the athlete voice and perspective directly into the research [14]. Semi-structured interviews allow participants to speak freely about their perceptions and experiences and are led by what is perceived most important to the interviewee [15]. Ethical approval was granted by the institutional research ethics committee (#2023/262).

### Researcher characteristics and reflexivity

The research team consisted of an athletic therapist with academic experience and lived experience as a Camogie player, and an athletic therapist and strength and conditioning coach. Their backgrounds provided valuable contextual insight, which informed the formulation of the research questions and interpretation of the data, but also carried assumptions about athlete wellbeing, progression and coaching practices. Although no prior relationships existed with participants, shared professional knowledge may have influenced rapport and interpretation. Reflexive strategies were used throughout the study to acknowledge and minimise potential bias, supporting the credibility and transferability of the findings.

### Participants

A total of fifteen adolescent Camogie players (14.4 ± 1.1 years) took part in a semi-structured interview. Participant demographic information is evident in Table 1. Participants had 7.2 ± 2.2 years Camogie playing experience (range: 3–10 years). All participants (n = 15; 100%) currently played up at least one age grade, with the majority playing solely at club level (n = 12; 80%). Most participants had played up an age grade previous to this current year (n = 10; 67%) and were involved in other sports alongside Camogie, with Gaelic football most common (n = 11; 73%).

**Table 1:** Participant demographic information.

Participant	Age (years)	Playing experience (years)	Currently playing up	First year of playing up	Current playing age grades^a^	Current playing levels	Current other sports played	Overall opinion of playing up
**1**	14	3	Yes	No	U14 + U16	Club	Gaelic football	Negative
**2**	14	4	Yes	No	U15 + U17	Club	Gaelic football	Positive
**3**	14	8	Yes	No	U15 + U17	Club	Gaelic football	Positive
**4**	16	5	Yes	Yes	U16 + U18	Club	Soccer	Positive
**5**	15	6	Yes	No	U15 + U16	Club + County	Gaelic football and Soccer	Positive
**6**	15	10	Yes	No	U16 + U18 + Adult	Club	Gaelic football	Positive
**7**	14	9	Yes	Yes	U14 + U16	Club + County	Gaelic football	Positive
**8**	13	7	Yes	No	U14 + U15 + U16 + U17	Club + County	Gaelic football, Irish dancing, horse-riding	Positive
**9**	14	9	Yes	No	U14 + U16	Club	Basketball	Positive
**10**	13	10	Yes	No	U14 + U15 + U16	Club	Gaelic football, soccer and hockey	Positive
**11**	15	9	Yes	Yes	U16 + U18	Club	Gaelic football	Positive
**12**	14	5	Yes	Yes	U14 + U16	Club	Gaelic football, rugby	Mixed
**13**	16	8	Yes	No	U16 + U18	Club	None	Positive
**14**	13	7	Yes	Yes	U14 + U16	Club	None	Mixed
**15**	16	8	Yes	No	U16 + U18	Club	Gaelic football, Badminton	Positive

^a^U14 = under 14 etc.

### Procedures

The interview guide was developed by adapting a similar guide utilised in previous research [6] and in line with the 5-step framework for the development of an interview guide [15], ensuring alignment with the research aims, identification of key topics, formulation of open-ended questions and sequencing for logical flow. The interview guide was composed of five main sections (full interview guide is available in S1 File). Section one collected participants demographic information and examined their Camogie experiences to date (e.g., “Tell me a little bit about how you got involved in playing Camogie”). Section two examined players’ perceived benefits of playing up (e.g., “What do you enjoy most about playing up an age grade?”), while section 3 examined their perceived negatives (e.g., “What do you dislike most about playing up an age grade?”). Section four explored the effects of playing up and how these perceived benefits and negatives influence players’ perceptions of playing up (e.g., “How do these perceived benefits you have discussed affect your experience of playing Camogie?”). The final section aimed to summarise participants’ experiences of playing up and explore their recommendations. A semi-structured format was employed to allow flexibility, with core questions addressing the central research objectives. In addition, a series of probing questions were incorporated to elicit further clarification and depth, enabling participants to expand on their experiences and ensuring the collection of rich, detailed data directly relevant to the study aims.

The interview guide was tested in an initial pilot study, where four semi-structured interviews were conducted with adolescent female Gaelic football players. This population was chosen for the pilot study due to the similarity in structure, terminology and developmental pathways across youth female Gaelic games, which allowed for effective evaluation of question clarity, relevance and flow in a comparable context. This approach also ensured that the Camogie player sample required for the main study was not compromised during piloting. The aim of the pilot study was to establish if the questions allowed players to convey their opinions adequately, and to facilitate training in research interview techniques for the moderator. Feedback on the pilot study was positive and no changes to the interview guide were required. The interview guide was subsequently reviewed and adapted to ensure that all language, terminology and examples were specific to Camogie before the main interviews were conducted. The results of the pilot study are not included in the main analyses.

Participant recruitment was facilitated by county executive committees, where a recruitment email was sent to the county secretary, which was subsequently shared with each of the clubs in that county. Recruitment was additionally facilitated through social media, with the recruitment poster shared online. A key element of the recruitment strategy was to ensure diversity of participants from urban and rural clubs across counties and provinces. Inclusion criteria specified that participants were required to be a youth Camogie player currently competing at an adolescent age grade at club level. While there was no requirement for participants to be playing up an age grade (in order to capture perspectives from both those who do and do not engage in this practice), notably all participants in the final sample were currently playing up an age grade.

Youth Camogie players meeting the inclusion criteria registered their interest, informed written parental consent and participant assent were provided online and the interview was scheduled at a convenient time. Participants were invited to have an additional adult present during the interview as per their personal preference. Information power guided participant sampling [16].

All interviews were conducted online through Zoom (Zoom Video Communications Inc., San Jose, California, USA) between May 1st and August 31st 2024 and were audio-recorded. All interviews were a minimum of 40 minutes in duration. An automatic transcription of the interviews was produced and the recording and transcripts were reviewed to ensure accuracy of the transcription. Participants were assigned identification numbers to maintain anonymity (e.g., P1 represented Participant 1).

### Data analysis and trustworthiness

All questions were answered by participants, and all interviews were included for analysis. Data were analysed using reflexive thematic analysis [17] via a six-step approach. Step one involved familiarisation with the transcripts, followed by coding of the relevant features of the transcripts in step two. Initial themes were generated in step three by grouping relevant codes, before the themes were reviewed for accuracy in step four. Step five involved defining and naming themes by reflecting upon all codes and collective patterns. The final reporting of results was produced in step six. Data analysis was an iterative process between both authors, who acted as “critical friends” to one another [18], to thoroughly explore and challenge interpretations of the data [17]. The Standards for Reporting Qualitative Research [19] have been adhered to.

## Results

Participants’ reasons for and perceptions of playing up an age grade, including perceived benefits and challenges, were identified. Additionally, participants’ recommendations for youth Camogie players considering playing up are presented, with a detailed summary of the identified themes, sub-themes and exemplar quotes provided in Table 2.

**Table 2:** Themes, sub-themes and participant exemplar quotes.

*Reasons for playing up an age grade*		
Theme	Exemplar Quote	
Personnel	The coaches … just cause they coach both the 14s team and the 16s and they asked a couple of us to play up into the 16s (P14) My dad is really supportive for Camogie. He’s always on me to get out and hit the ball and that he goes to all my matches so I think he was important for me deciding to play up (P6) My sister did it as well, she’s older than me. She’d be playing with the senior team now, but when she was underage, she played up. So, I saw her do it so I just thought, oh yeah, it’s not that big a deal (P13)	
Autonomy	Just myself … I wanted to give it a try (P15)	
Experience and opportunity	Just thought like it would a good opportunity. Just, you know, like I said, I’m quite competitive and it’s a challenge (P13)	
Number shortage	You have to be there because sometimes, we actually might have only one or two people on the bench. We might have 16 or 17 girls, so you can’t just not show even though it might be tough (P12)	
Individual skill level	I’ve always been pretty good at Camogie, especially the last few years, and then last year there was one or two games where I was asked to come up, but I didn’t think I was ready. I was very young, it was my first year at 16s so I thought I was maybe not confident enough. This year, when I was asked initially like closer to the start of the year, I had a lot more confidence in myself and I thought I was a better player, and that I should definitely give it a go. So I just thought, why not give it a try (P11)	
*Perceptions of playing up an age grade*		
**Theme**	**Exemplar Quote**	
Initial challenges	At the start they didn’t really know who I was and they’re like ‘Oh, what’s she doing here? She’s a good bit younger’. So they were kind of keeping it to themselves, but after a while they were like, ‘Okay, she’s not bad’ so now they pass the ball (P8) The physicality. Those girls are essentially 2 years older. They hit harder so there’s definitely an extra few bruises and bumps, which at the start is a little tougher. It’s not nice being smaller, coming out of training and being bruised and stuff, but that was probably the worst thing about it, but you get used to all that stuff quick enough (P11)	
Differences during training and matches	It’s also quicker, the girls are just quicker and have improved their skills more. Their catching and passing is just crisper, It’s quicker, if you make a mistake, you’re more likely to get punished. At 16s, you know if you don’t catch your ball cleanly and you don’t pick you up straight away you might get away with it, whereas at minors, if you don’t catch it straight away off a pass, and if you’re not crisp, or quick to correct it, you’re going get turned over nearly every time. You’ve got to be quicker, you’ve got to make less mistakes so really it’s been just reducing your mistake count and just trying to keep up with the play (P4)	
Differences in physical characteristics of other players	It’s always just faster and more physical. Whenever you play up an age, cause even like between 16s and 18s, the girls aren’t really taller, but they’re just more physical, and they’re just stronger. The girls are stronger, and it’s a little quicker. You have to just make the right decisions or it all goes wrong (P13)	
Playing up but preference for playing own age grade	I prefer the 16s cause it’s all my friends from school and all the way up. I played them since like under-8s, and under-7s and under-9s so I probably prefer playing with the 16s, but the minors is always good craic with the girls (P11)	
*Perceived benefits of playing up*		
**Theme**	**Sub-theme**	**Exemplar Quote**
Competence as a Camogie player	Performance enhancements	When you go into a team where obviously all the girls are older, bigger and quicker and they’re all really good, you improve, you have to. It’s either sink or swim. You have to improve and you have to get better otherwise you may not play (P4) It’s faster pace and it’s definitely higher standard I guess, you have to improve your game to play at that level. After a couple of months now I’ve definitely caught up, and I’m playing at that level and then when I play the 16s games, I feel like I’m one of the better players now, definitely, because I’m used to the minors (P11)
	Skill development	More skills and just the basic drills, things to do when you’re on the pitch. So just overall I feel like I am kind of broadening my horizon with regards to my skills. Little tips, little tricks are good (P9)
	Exposure and experience	I get to play more, which you know, I love playing Camogie … I am getting to play as much as possible, which is class (P4) I played halfback for them as well, which is also different space and speed. I get to try out different things because of the different positions which is good (P10)
	Benefit to other sports	Yeah, it’s made me a lot more physical outfield in football as well, because in Camogie I’m usually in goals, but for under-16s I play backs or forwards, which helps when I’m going in for a ball or something like that for football as well (P7)
	Coaching and playing standards	Just the calibre of coaching. Honestly like the difference between the teams could just be the people on the team as well, or the calibre of players. … but the coaching standard is better I think (P6)
Psychosocial competence	Social gains	Getting to know some of the other girls is always good … I’ve probably a few more friends. I’m going to be close to some of the older girls now, which is always good. Having more friends around is always great (P4)
	Resilience	When I played before, sometimes I doubted myself. Then playing with the older team as I got more confident with them, I was even more confident on my team, so I got better for my own team (P2) Just figuring it all out. Kind of seeing what’s going on, and pushing my limits. Going a little bit further than I’m used to going (P7)
*Perceived negatives of playing up*		
**Theme**	**Sub-theme**	**Exemplar Quote**
Pressures and demands	Pressure to play and perform	They’re always saying the passes have to be straight to the hand … if I would mess up a pass and it goes far away from the player, then I feel pressure on myself. I’m like ‘oh the coach, I know he’s going to be disappointed’ (P3) You just don’t want to have a bad game, because, you know, you might be a bit more nervous than at your own age group, because it might be more likely you’re going to have a bad game because you’re playing up (P12)
	Competing demands	**Clashes between sports and age grades**
		Because some of my friends in school are on my under-16 team, they’d be a bit disappointed when I don’t go training with them or if I miss a match with them to go and play up (P6)
		**Excessive demands**
		The 14s play on Sundays usually so if I have a match with the 16s on the Friday or Saturday, sometimes I feel really sore going into the 14s match. I feel like sometimes I’m not playing as well because of it (P12)
		**Negative effect on school**
		At the weekends, you have two matches so I just have to make sure I get my homework done on Friday evenings. If I’ve had two matches over the weekends, I could be too tired to do my homework (P11)
	Increased injury risk	Some girls are bigger and stronger, and a lot of girls I know, they get hurt from playing up age groups when they first go up and play an older age group and they’re not ready for it (P8)
Adverse peer interactions	Social issues	Sometimes it puts me off because you’re playing with people who you don’t really know and they don’t really want to get to know you so sometimes it would actually turn me off playing (P1) Any younger girls who weren’t called up from your team, they’re jealous of you, and they won’t talk to you or anything after you’re being called up (P8)
	Lack of trust from older players	Sometimes I feel like they don’t pass to me because they don’t trust me (P14)
Impaired intrinsic motivation	Reduced confidence	Having low confidence or bad confidence really affects how you play in your next match. You need to have a good head, be thinking good in order to play well. So if you have a bad game playing for the older girls, that can make you play bad in the next game, at your age or playing up (P3)
	Lack of enjoyment	It feels like a chore … you kind of just show up, get the match over with and then head home instead of hanging around about afterwards (P1)
*Recommendations for youth Camogie players considering playing up an age grade*		
**Theme**	**Sub-theme**	**Exemplar Quote**
Would you recommend playing up an age grade?	Yes	I definitely would encourage people to do it. I definitely would recommend them to play up because you’re going to learn more, you might make more friends, you might become a better player, just as I have. I definitely have done. When you become a better player, then obviously it’s going to be more enjoyable (P4)
	Unsure	I’m not sure, it depends. If you’re good yeah, but if you’re particularly small then no. I know in other clubs girls from my age are playing 16s and minor. I’m lucky I’m only playing 16s, mostly for the second team, but I wouldn’t want to play with the minor team. No way, that’s just too physical, 16s is fine. So I would say it all depends on the person themselves and how physical they are really (P12)
Recommendations	Social inclusion or team bonding	We had a team bonding thing at the very start of our season. I think with the first two or three training sessions, if we had more team bonding, getting to know and getting to trust each other, then the actual proper training. We’d do hand passing or strikes or you go in a group of four and you go around and you’d kind of see how many you get and you switch partners every time, a new person. Then one of the other ones where when we do our first like two laps of the pitch, you would be paired up with a new person every time and you had to get two or three facts about them (P2) Just make an effort to speak to everyone. Speak to everybody and include everybody. You don’t have to be best friends with them, but you know just include them. Be friendly (P5)
	Support and encouragement	**From coaches**
		Be patient with them at the start, they’re bound to make a couple of mistakes in training or in games but just be patient. They are obviously good players because they’ve been asked to play up, you’re not just picking anybody so just be patient, and they will learn and they will get up to speed (P11)
		**From older players**
		Just keep on being understanding if someone doesn’t get this skill right the first time or takes a little bit longer than the rest of the team to get it right. Just know that it’s a younger person and they’re not going to get it right the first time, maybe not the second or third time either but just keep on being supportive (P6)
	“Just do it”	Always try it and if you don’t like it you can stop, but always try it (P2) It will improve you so much, not even just as a player, but as a person. I definitely become more aware of myself, my personality and everything, from speaking to older girls about how they play and what the best way to play is, so you feel like you’re just improving all the time (P6)
	Autonomy	Don’t just do it because you’re asked. I actually am really glad that I said no last year, cause I didn’t think I was ready, but this year I was ready to give it a go. It’s still a learning curve but I don’t think I would have been able for it last year. I’m glad I went up this year, so I would say if you think you could give it a decent shot, give it a go (P11)
	Prepared to play up	Maybe if I was a bit bigger, it might help. Maybe if I was just bigger and faster, it would help so maybe starting gym work earlier could be good (P14)

P1 = Participant 1 etc.

### Reasons for playing up an age grade

Coaches, parents, friends and family significantly influenced youth Camogie players’ decisions to play up an age grade. Most participants discussed that within youth playing grades, there is a prevalence for coaches to be involved across several teams, allowing them to identify and encourage capable players to step up. Parental support was also integral, while friends and family, such as siblings or cousins who have previously played up, make the transition feel natural and less daunting. Beyond external influences, the experience and opportunity to play up and player autonomy were key factors, that although invited to play up by their coaches and encouraged by their parents, the final decision came back to them, allowing the viewpoint of “*if I wanted to, I could*” (P7). Several participants also acknowledged they compete in older age grades due to insufficient player numbers, necessitating their participation to field a full team, creating an implicit pressure to step up and ensure team viability. Despite this external expectation and sense of obligation, participants felt their individual skill levels were sufficient to excel at the higher level.

### Perceptions of playing up an age grade

Playing up an age grade presented initial challenges. Socially, integrating into an older team was challenging, as established players were perceived to initially be reluctant to trust or involve new, younger members. As their ability and reliability are proven, social barriers break down, leading to greater acceptance. Younger players also faced initial physical challenges, such as increased intensity, physicality, faster pace, and higher skill levels. Adapting to the more demanding and physical nature of matches can be frustrating but it was perceived “*you get used to all that stuff quick enough”* (P11).

Participants also noted key differences exist during training and matches when playing up that persist beyond that initial period. The game demands a faster pace, quicker ball movement, and increased physicality, meaning you “*have got to be on your toes a bit more*” (P5) as you “*get less time to do anything*” (P14). Acknowledging these differences exist was not perceived as a negative but rather that “*it’s just a better level really overall*” (P15), which players must be aware of and adapt to when playing up. However, it was perceived as a negative that it can be “*too crowded sometimes*” (P5) when two age grades train together and that results in less playing time and more “*waiting for the lines in the drills*” (P5). Although expressing a desire to still play up an age grade, some participants preferred playing at their own grade.

### Perceived benefits of playing up an age grade

#### Competence as a Camogie player.

Participants perceived significant performance enhancements and accelerated skill development from playing up that built physical strength and improved their tackling and scoring abilities. Many participants noted that when they return to their own age group, the game feels easier and their improved physicality and confidence makes them braver in tackles, stronger in challenges and feeling like they have “*stepped up to the plate”* (P4). Playing at a faster pace also improved decision-making, ball-handling and passing accuracy, forcing quicker reactions. These benefits not only impacted current performance at their own age level but it “*gets you ready for the next year*” (P9). Participants also perceived that playing up provided exposure to more structured training and a higher calibre of coaching, including strength and conditioning, opportunities which they believe are not provided at their own age grade. Many participants viewed this exposure as a valuable way to improve and to “*get more out of it*” (P7). The increased exposure diversified their experience and allowed them to try different positions and play with a variety of teammates, with the benefits also extending to other sports.

#### Psychosocial competence.

Social gains: The camaraderie of playing up and making new friends and connections was perceived to strengthen players’ sense of belonging. The mentoring role of older players was noted, most notably that they “*teach you stuff that you wouldn’t have been taught in under-14s*” (P9) and they “*can help you out as well, if you’re struggling with anything*” (P8). These new fostered relationships with older players often extend beyond the playing field, where players “*know more of the girls at school*” (P13). The longevity of these relationships was deemed valuable, especially when transitioning to senior Camogie and winning was more enjoyable when shared with a mix of familiar and new teammates. Being part of a team and playing with the same group of girls where “*no one’s ever putting pressure on each other*” (P8) was important and facilitated greater fun and enjoyment.

#### Resilience.

Participants noted playing up an age grade boosted players’ self-confidence and was recognition of a player’s talent, hard work and abilities. Players feel “*appreciated more when you play up*” (P6), where it is “*an honour in our club*” (P10) to be asked to play up. The challenge and opportunity to push yourself, along with the opportunity for maturity and personal growth were additional perceived benefits.

### Perceived negatives of playing up an age grade

#### Pressures and demands.

Many participants stated, *“you’re there to help [the coaches] out if they’re struggling for players”* (P1) but as a result, every mistake felt magnified as they are viewed as the “*best players*” (P11). This mindset led to overthinking, making it harder to play naturally and confidently. This was exacerbated by the internal expectation and “*putting that pressure on myself to perform*” (P5). Although perceived as a negative, the pressure to perform benefitted performance, where *“it just kind of spurred me on and pushed me to keep going, keep practicing, to really want to be better*” (P4).

Competing demands and clashes between sports and age grades also made it difficult to play up. There can be conflict between game schedules, where matches at different levels overlap, resulting in frustration from coaches and teammates, especially if a player is unavailable for their own age-grade games or trainings. With these clashes, participants describe a feeling of “*pulling from different sides*” (P6) and peer frustration. The evident clashes can also place excessive demands on youth Camogie players, where their performance is hindered or they feel pain when playing. The negative effect on school of playing up was also considered. Tough training sessions or matches makes players struggle to get up in the morning and it can be difficult to find time for homework when playing in different matches.

Participants acknowledged an increased injury risk when playing up. The risk was perceived to be even greater when moving up to adult grades, where opponents can vary widely in age and strength, especially if a player is not physically ready. Beyond the physical risks, there was also a mental toll, with anxiety and fear about getting injured.

#### Adverse peer interactions.

Social issues with older girls impaired the experience of playing up. The older players often had their own established friendships and team dynamics, making it hard for younger players to feel included, leading players to “*dread it a bit more*” (P1). A lack of trust was also noted as their older teammates would prefer to play with peers their own age, negatively affecting their Camogie skills. There were also issues evident with their same-aged peers when they were called up ahead of their friends and teammates.

#### Impaired intrinsic motivation.

Playing up an age grade resulted in reduced confidence for young Camogie players, especially when performance was affected. A poor performance when playing up could carry over into their next game, making them more hesitant and unsure of themselves. Participants also noted that playing up at times is not enjoyable and sometimes “*feels like a chore*” (P1) and “*takes a bit of fun out of the sport*” (P1), which can negatively affect their mood.

### Recommendations for youth Camogie players considering playing up an age grade

Most participants overwhelmingly stated they would recommend playing up. It was described as a “*good experience”* (P11) and an opportunity that can help you “*feel more comfortable and confident”* (P11). Some participants, however, were unsure, acknowledging that “*the team would probably still need numbers*” (P14), but playing up was dependent upon players’ physical characteristics; “*if they’re big and fast enough and they want to give it a go or try it, but if they are small, maybe not*” (P14).

Based on their own experiences, recommendations for those considering playing up were provided. Firstly, participants recognised a need for strategies to support social cohesion to make young players feel welcome and part of the team. Participants recommended team bonding activities and for older players to bring the new younger team members into the group, chat with them and offer tips to help them improve. Support and encouragement from coaches was also recommended, with participants consistently acknowledging that it is important for coaches to be patient and give young players time to settle in. Most importantly, participants recommended that the coaches “*make them feel included and listen to them*” (P10) and make them feel valued for being there.

Many participants recommended that young players “just do it” when considering playing up because if they are asked to play up, they have demonstrated proficiency at their own age grade and are therefore good enough to embrace the challenge of playing up and should “*just give it a go. You’ve nothing to lose*” (P13). However, autonomy was also acknowledged, where players should “*do it because you want to* *do it”* (P12) and they believe they are good enough and prepared to play at that higher standard.

## Discussion

The current study provided a qualitative investigation of youth Camogie players’ perceptions of playing up an age grade. Given that almost one in six youth Camogie players dropout of the sport [2], the common practice of age-grade progression, or playing up, warrants examination. It is imperative to integrate the athlete voice into this research to comprehensively ascertain their lived experiences regarding participation in higher age categories. When interpreted through the lens of the Self-Determination Theory [20,21], the findings highlight the influence of the playing environment on youth players’ intrinsic motivation to play up and participate in Camogie. Although initial challenges were reported, 80% of participants expressed overwhelmingly favourable perceptions of playing up. Most notably, benefits were in relation to their Camogie skill development and psychosocial competence, outcomes that strongly satisfy their need for *competence* as a Camogie player. Despite these benefits, youth Camogie players perceive associated pressures and demands of playing up, adverse peer interactions and impaired intrinsic motivation as negatives of playing up, which actively hinder their need for *autonomy* and *relatedness*.

Youth Camogie players believed playing up an age grade significantly enhanced their competence and overall performance. Participants articulated a sense of accelerated development, which was directly attributed to the increased exposure and experience gained by playing up. This includes greater playing time and access to elevated coaching and playing standards, all of which are instrumental in fostering player development. Improvements in sport-specific skill [6] and technical and tactical performance [9] have also been previously observed among youth athletes playing up an age grade. The enhanced learning environment can allow players to be consistently pushed beyond their comfort zones when playing up, leading to rapid skill acquisition and tactical understanding [22]. The necessity to perform at a higher level against more developed athletes can be a catalyst for individual improvement, directly contributing to the development of physical (e.g., improved fitness, motor skills) and cognitive assets (e.g., enhanced decision-making, tactical awareness) [23]. This mastery and perceived skill development can directly impact a player’s confidence and enjoyment, reinforcing their motivation [24] and commitment to the sport [25]. Research in Gaelic games has shown that emphasising the development of key bio-motor qualities (e.g., strength, speed, power), while also creating developmentally appropriate and autonomy-supportive coaching environments can facilitate holistic player development [26].

Participants also perceived a valuable transfer of these enhanced performance attributes to other sports. This suggests the cognitive and physical adaptations required to succeed when playing up are not sport-specific but contribute to broader athletic development. The concept of multi-sport participation in youth development is supported, where skills learned in one domain can positively influence performance in another, contributing to a more holistic athletic foundation [12]. In particular, the development of athletic motor skill competencies [27] through Gaelic games participation can support the development of perceptual and decision-making skills applicable to other sports [26]. This transferability underscores the development of robust physical and cognitive assets that are not confined to a single sport.

Camaraderie, fun and enjoyment, and the development of resilience were also key psychosocial benefits of playing up an age grade in Camogie, which have also been identified in previous research [9]. Performance improvements are facilitated by positive and enjoyable practice activities [28], while a well-designed learning environment that appropriately challenges skill and game development can enhance group connectedness through stronger relationships with coaches and teammates [29]. The experience of more positive social engagements with teammates also enhances feelings of identity and perceptions of belonging [30]. The connections youth athletes form with their teammates, both in how often they happen and how meaningful they are, are foundational to their social and moral development [30]. The development of these emotional assets (e.g., resilience and confidence) can be fostered when playing up, as players learn to cope with higher pressure environments, and social assets through enhanced communication and teamwork within a more experienced group [23], leading to higher levels of sport enjoyment [31].

Overall, improved competence as a Camogie player and enhanced psychosocial competence resonate strongly with the Personal Assets Framework (PAF; 21,26). The PAF proposes youth sports experiences are built upon three dynamic factors; the types of activities youth participate in, the physical environment and the social setting. When these components are well-designed and interact effectively, their interplay creates positive sport experiences, leading to the development of confidence, competence, connection and character. Ultimately, this contributes to long-term participation, personal development and enhanced performance [23]. Given the recommendations provided by participants to future youth Camogie players considering the option of playing up, these three elements of the PAF must be fundamentally integrated into coaching practices in Camogie to maximise these potential benefits. In addition, an autonomy-supportive coaching environment that promotes player autonomy, competence and relatedness should be fostered to positively influence the sport experience, improve performance and develop long-term satisfaction in all three Self-Determination Theory basic needs [29,32].

Sport is assumed as inherently positive, automatically generating positive outcomes for those individuals and communities that engage with it [33], an assumption that could be extended to youth Camogie players. However, despite evident benefits, for many youth Camogie players, playing up was associated with significant pressures and demands from their coaches and teammates. This often led players to feel overwhelmed by the heightened expectations of the older age group. Previous research in youth sport has noted the expectation for players to show their worth to their older teammates and play well to avoid losing their trust [6]. Perceived pressure from coaches has also been demonstrated, where mid- (14–17 years) and late-stage (17–20 years) adolescents report higher perceived coach pressures compared to early-stage (<14 years) adolescents [34]. Pressure can be detrimental to performance through overconcern for mistakes or self-doubt [35], but youth athletes must be able to withstand the pressure and learn to use it to their advantage to elicit improved performances [36]. Given that the developmental pathway of youth sport participants is influenced by the quality and characteristics of their coaching and support networks [37], the recommendation of support and encouragement from coaches must be fundamentally considered to maintain youth participation in Camogie. This also emphasises the importance of autonomy-supportive coaching environments in Gaelic games [26] to ensure athletes’ psychological needs for autonomy (choice), competence (skill development), and relatedness (social connection) are effectively met [38].

Furthermore, the players’ narratives highlighted the struggle with competing demands between sports, a challenge exacerbated by the increased commitment required for the older age group. This aligns with findings by Brenner et al. [39], that showed the time-consuming nature of elite youth sport can lead to stress and a reduction in engagement with other life activities. In addition, youth athletes who participate in more hours of sport per week than their age in years, or whose organised sport versus free play time ratio exceeds 2:1, are at increased risk of serious overuse injury [40]. This perceived increased risk of injury was a significant concern. The unique features of the youth sport environment and their influence on lifelong musculoskeletal health and physical activity behaviour are well documented [41] and are supported by physiological literature, which notes younger athletes, who may not have reached skeletal or muscular maturity, are at a greater risk of overuse and traumatic injuries when competing against physically larger and stronger opponents [42–44]. In addition, reductions in flexibility, coordination and balance during the adolescent years, can increase youth athletes’ susceptibility to injury, leading to potential psychosocial disturbances, such as mood swings, stress, anxiety, depression and social pressure, in addition to isolation and disengagement from their friends [45].

There is a clear need for coaches, parents and organisational leadership to recognise that a uniform approach to playing up an age grade is insufficient. Policies should instead incorporate individualised considerations to account for highly variable individual maturation rates and the diverse physical, psychological and developmental needs of young players [26,46,47]. This player-centred ethos aligns directly with the Gaelic Games 2030 Vision [48], which mandates a scientifically informed approach to maximising player potential and retention. Bio-banding, a strategy that groups athletes based on biological maturation rather than chronological age [49], should be considered. However, bio-banding must be grounded in objective measurements, as solely relying on the coaches’ subjective perspective is an invalid method for estimating biological maturation [50]. It must also be flexible, as rigid grouping risks excluding late-maturing talent [51]. Evidence supports the benefits of such approaches, with youth soccer players reporting that maturity-matched environments are both developmentally and psychologically rewarding [52]. Further research specific to Gaelic games is required.

The social dynamic within the older team was another critical source of negative experiences. Participants reported a lack of social cohesion with older girls, feeling like outsiders and struggling to integrate into an established social group. This is a common phenomenon in team sports, where social hierarchies can develop based on age and skill level [53]. The perceived lack of trust from older teammates was particularly damaging, as players felt their abilities were doubted, which negatively impacted their sense of belonging and their willingness to take risks on the pitch. This a breakdown of the relatedness and social fabric of the team, which is a key predictor of positive youth sport experiences and continued participation [54,55]. A lack of connection with a supportive peer group has been identified as a contributing factor to youth sport dropout [56], particularly when participation demands conflict with the social developmental needs and priorities of young athletes [57]. Youth sport environments fostering both task and social cohesion can facilitate greater participation experiences for athletes, while also demonstrating benefits for personal, social and cognitive skills, and goal setting [58]. This warrants particular attention from coaches in teams where girls are participating in an older age grade.

The combined effect of performance pressure and social isolation directly impacted players’ intrinsic motivation. Players expressed feelings of self-doubt and a diminished sense of self-worth when they were unable to meet the higher standards or felt they were not contributing effectively to the team. Perceived self‑efficacy has been acknowledged as a significant moderating variable influencing the extent to which young participants reported positive experiences in sport [59]. Sport participation can contribute to self-confidence through encouragement, self-talk and successful performances [60]. Working with youth Camogie players requires awareness of the developmental and psychosocial factors influencing their sporting experiences, with coaches playing a key role in fostering environments that support autonomy, competence and relatedness.

### Practical implications

These findings have clear practical implications for stakeholders involved in youth Camogie. The strong player perception of enhanced competence and skill development when playing up an age grade carries significant practical implications for the development of youth Camogie. Firstly, it highlights the importance of creating challenging environments that motivate young athletes to grow. For example, coaches could utilise small-sided games with specific technical and tactical constraints, implement positional rotation within high-decision-making central zones (midfield, half forward/back), and prioritise process over outcome-oriented goals. While safeguarding against burnout and injury is paramount, providing opportunities for players to test themselves against higher standards can be beneficial. This environment must be underpinned by autonomy-supportive coaching practices. Consequently, coach education should shift from rigid, prescriptive frameworks regarding *what* to coach toward a facilitative approach focused on *how* to coach [29], while also providing education on training load management, burnout and the psychosocial aspects of youth Camogie participation.

Secondly, coaches should consider individual readiness and psychological maturity, not just chronological age or number shortages, when making decisions about player participation. Identifying players who thrive in more challenging settings and offering them appropriate opportunities could optimise their development. Coaches can assess readiness by evaluating emotional regulation, self-efficacy and social adaptability through structured trial exposures, collaborative feedback and regular player check-ins. Crucially, these practices must be underpinned by an autonomy-supportive and psychologically safe environment that prioritises player learning without fear of making mistakes. It is also imperative that coaches are proactive in creating an inclusive and supportive team culture that actively bridges the age-related social dynamic. This could involve structured team-building activities or the provision of targeted support and mentorship for younger players.

Finally, understanding players’ perceptions of their own development is critical. Fostering a belief in their improving competence can be a powerful motivator and may contribute to sustained participation and performance, aligning with the holistic development promoted by the Personal Assets Framework. This could be facilitated through incremental exposure to playing up, beginning with phased integration into training sessions before introduction to competition.

### Limitations

This study solely considered the personal perspective of players currently playing up but not those who wish to play up but are not selected, or those adversely affected by younger teammates playing up. In addition, given the prominent role of families in youth Camogie, and the common situation in which parents act as coaches, participants may have been hesitant to disclose negative experiences. This dynamic may also have introduced social desirability bias, leading some athletes to present more favourable accounts of playing up an age grade. Future research should ensure an inclusive sample of youth Camogie players that are both playing up and not playing up to ensure the voices and perceptions of all players are considered. The study was also limited to the perspectives of youth players; therefore, future research should incorporate the views of coaches and parents to provide a more comprehensive understanding that reflects all relevant stakeholder perspectives. The findings are also context-specific and may have limited transferability to other sports or youth populations.

## Conclusion

This study offers important insights into the perceived benefits and challenges associated with playing up an age grade in youth Camogie. Players identified psychosocial competence and enhanced performance and skill development as key advantages, highlighting the developmental potential of playing up when appropriately supported. However, participants also acknowledged notable risks, including heightened physical and psychological demands, increased injury vulnerability, and potential disruptions to peer relationships and intrinsic motivation. These findings underscore the need for individualised decisions around playing up that balance opportunity with athlete welfare. Coaches, parents and sport organisations play a critical role in facilitating safe and positive experiences by ensuring that players’ readiness, motivation and autonomy are central to the process. Overall, while playing up can provide meaningful developmental gains for youth athletes, its successful implementation requires a holistic, player-centred approach that mitigates risks and supports long‑term engagement in sport.

## Supporting information

S1 File Interview Guide. (DOCX)
