# Supplementary material for: Stepping up: A qualitative examination of youth Camogie players’ perceptions of playing up an age grade
Source: PLoS One. 2026 Aug 11;21(8):e0355563. doi: 10.1371/journal.pone.0355563 (PMC13460593; doi:10.1371/journal.pone.0355563)
Supplement: Supplementary file 1 [file pone.0355563.s001.docx]

**S1 File – Interview Guide**

**The interview guide has been adapted from previous research conducted by Goldman et al. (2022) in line with the 5-step framework for the development of an interview guide designed by Kallio et al. (2016).*

**General Introduction**

Thank you for agreeing to participate today to talk about your perceptions and experiences of playing up an age grade within Camogie. We want to understand what playing up looks and feels like to you, and how it affects you.

This is *your* perception of playing up an age grade. This is unique to you so there are no right or wrong answers. This is an open opportunity for you to discuss how you feel about it. Anything you discuss is completely confidential and will not be linked back to you in any way. If you don’t want to answer a question, you don’t feel comfortable or you want to stop at any time, that is not a problem. You are free to do so, and this will not affect your relationship with the Camogie Association, with DCU or with anyone else. Is that okay with you?

Finally, just to confirm again, this interview will be audio-recorded so just with the sound. This will be used to clarify the transcription of our interview only and it will not be shared. It will be deleted once the transcription has been reviewed. Is this okay with you?

Before we start do you have any questions for me?

Are you happy to start the interview?

I will start the audio recording now. *[Start the audio recording if the player consents.]*

I will now ask you to confirm your consent to participate by saying: “I consent to participate in this study.” [*Proceed if the player consents.]*

**Participant Introduction**

I would like to start by asking you a couple of questions about your sport background:

*Brief Demographic Information:*

- Can you tell me what age you are?
- Do you play Camogie at club or county level currently?
- What age grade or grades do you currently play?
- How many years have you been playing Camogie in total?
- Do you currently play up an age grade in Camogie?
- Have you previously played up an age grade in Camogie?
- Do you play other sports currently?

*Brief Introductory Questions – the Camogie experience:*

- Tell me a little bit about how you got involved in playing Camogie.
- What do you enjoy most about playing Camogie?
- You mentioned you currently play at __________ level, how does it make you feel to be a member of this/these Camogie team(s)?

**Benefits of Playing Up**

We are going to start talking about your perceptions of playing up an age grade with Camogie. When we talk about ‘playing up an age grade’, we are describing the period of time where a player plays a chronological age grade above the current primary age grade for that player. So for example, if you are 14, you may play at under-14 and at under-16 levels. Do you understand?

So firstly, I want to ask, what do you think are the benefits of playing up an age grade in Camogie?

*Probing questions:*

- *What do you enjoy most about playing up an age grade?*
- *How has playing up an age grade been beneficial for you?*
- *Does playing up an age grade benefit you (or others) in terms of being a better Camogie player?*
- *Does playing up an age grade in Camogie benefit you (or others) in other sports?*
- *Does playing up an age grade in Camogie benefit you (or others) in school?*
- *Why did you make the decision to (not) play up an age grade in Camogie?*
- *If you had to make the decision again now, would you still choose to (not) play up an age grade in Camogie?*
- *Who was the most important person in making the decision to (not) play up an age grade in Camogie?*

**Negatives of Playing Up**

Now, what do you think are the negatives of playing up an age grade in Camogie?

*Probing questions:*

- *What do you dislike most about playing up an age grade?*
- *What are the cons / downsides / drawbacks of playing up an age grade in Camogie?*
- *How has playing up an age grade been detrimental for you (or others)?*
- *Does playing up an age grade negatively affect your ability (or others ability) to be a good Camogie player?*
- *Does playing up an age grade in Camogie negatively affect you (or others) in other sports?*
- *Does playing up an age grade in Camogie negatively affect you (or others) in school?*
- *If you had to make the decision again now, would you still choose to (not) play up an age grade in Camogie?*

**Effects of Playing Up**

How do these perceived benefits you have discussed affect your experience of playing Camogie?

*Probing questions:*

- *You mentioned benefits of playing up, such as ______, do you think these benefits make playing Camogie a better experience?*
- *You mentioned benefits of playing up, such as ______, do you think these benefits negatively affect your experience of playing Camogie in any way?*

How do these perceived negatives you have discussed affect your experience of playing Camogie?

*Probing questions:*

- *You mentioned negatives of playing up, such as ______, do you think these negatives make playing Camogie a better experience?*
- *You mentioned negatives of playing up, such as ______, do you think these negatives negatively affect your experience of playing Camogie in any way?*

How do these perceived benefits you have discussed affect your development of skills that are specific to Camogie?

*Probing questions:*

- *You mentioned ______ as benefits of playing up in Camogie, do you think these benefits support your development of specific Camogie skills and make you a better player?*
- *You mentioned ______ as benefits of playing up in Camogie, do you think these benefits negatively affect your development of specific Camogie skills?*
- *By playing up an age grade, the development of skills important for Camogie can be either positively or negatively affected by these benefits. Can you tell me what you think?*

How do these perceived negatives you have discussed effect your development of skills that are specific to Camogie?

*Probing questions:*

- *You mentioned ______ as negatives of playing up in Camogie, do you think these negatives support your development of specific Camogie skills and make you a better player?*
- *You mentioned ______ as negatives of playing up in Camogie, do you think these factors negatively affect your development of specific Camogie skills?*
- *By playing up an age grade, the development of skills important for Camogie can be either positively or negatively affected by these negatives. Can you tell me what you think?*

**Wrapping Up (Recommendations)**

We are just coming to the end of our chat now so just a few more questions.

- Looking back, what would have made your playing-up experiences better?
- What advice would you give to other Camogie players who may be thinking about playing up?
- What advice would you give to the coaches of Camogie players who play-up?
- What advice would you give to the teammates of Camogie players who play-up?
- Would you recommend playing up to other Camogie players? Why or why not?
- Overall, what did you find different about the sport of Camogie when playing up an age grade?

**Interview Conclusion**

- Summarise – So we have spoken a lot about _____
- Reflect – Have I understood correctly or is there anything I have missed?
- Opportunity – Do you have any final thoughts about playing-up that you feel are important and that we did not already cover? Do you have any questions you would like to ask me?
- Thank you – We will end the discussion here. Thank you very much again for participating and sharing your thoughts and feelings about playing up
