# Supplementary material for: Stepping up: A qualitative examination of youth Camogie players’ perceptions of playing up an age grade
Source: PLoS One. 2026 Aug 11;21(8):e0355563. doi: 10.1371/journal.pone.0355563 (PMC13460593; doi:10.1371/journal.pone.0355563)
Supplement: Supplementary file 3 [file pone.0355563.s003.docx]

**PONE-D-26-14972**

**Stepping Up: A Qualitative Examination of Youth Camogie Players’ Perceptions of Playing Up an Age Grade**

We would like to thank the reviewers for their time and consideration of our manuscript. The comments were incredibly helpful and insightful. We have considered the comments and specific feedback provided and have made considerable edits. We hope the edits we have made are sufficient in making this manuscript suitable for publication. Please see below for comments specifically addressing each reviewer comment.

**Reviewer #1**

**General feedback:**

Thank you for the opportunity to review this paper. This area of underage Camogie is under researched, and this information could potentially add to the field. This is a very well written piece of work and the authors should be commended for their input into the paper.

Thank you for your kind comments.

There is still some work required to ensure you are maximizing the informative results you have extrapolated from the data. There is an opportunity here for you to provide recommendations to the GAA and coaches from the best practices from coaching science and sports psychology. This could be an important practical application of your research for coaches/practitioners to prepare youth Camogie players to stream & play up.

Thank you for this comment. We have updated the Practical Implications section to maximise the impact of our findings in this area.

The results section is far too long, and I’ve provided recommendations and feedback in the comments below. The results section is far too long, please specific feedback below on streamlining this section.

We have significantly edited the results section based on this comment and the feedback provided below. We hope the edits have addressed your concerns.

The authors should refine their discussion section before the manuscript can be reconsidered. The discussion section makes broad recommendations; this needs to be specific to your findings with support from the sports science and coaching science literature (See discussion section below).

We have edited the Discussion section to provide context to our findings utilising the available related research. We hope this addresses your concerns.

The authors may consider incorporating the concept of bio-banding, physical development into the discussion section using the following research papers:

- Fitzgerald, F., Campbell, M., Kearney, P. E., & Cumming, S. P. (2025). The eyes don’t have it: Coaches’ eye is not a valid method of estimating biological maturation in male Gaelic football. International Journal of Sports Science & Coaching, 20(1), 35-44.
- Kearney, C., Coughlan, E. K., O'Connell, A., Lacey, S., & Burns, C. (2025). Bigger but are they better? Investigating growth, maturation, and physical performance metrics in underage Gaelic games development squads. The Journal of Strength & Conditioning Research, 39(6), e806-e814.

Thank you for providing these references for consideration. We have incorporated the literature into the manuscript.

Under-researched area in GAA and especially in Camogie. Prof Sean Cumming also has some research on players opinions on playing up in the academy football could be included in the discussion:

- Bradley, B., Johnson, D., Hill, M., McGee, D., Kana-Ah, A., Sharpin, C., ... & Malina, R. M. (2019). Bio-banding in academy football: player’s perceptions of a maturity matched tournament. Annals of human biology, 46(5), 400-408.

Thank you for directing us to the work of Prof Sean Cumming and this paper from Ben Bradley. We have incorporated their work into the manuscript.

There are some minor formatting issues throughout the document, please ensure they are addressed before re-submission.

Thank you for highlighting this. We believe these issues have now been addressed but please direct us to anything we may have missed.

Concerningly, some of the seminal research in GAA, streaming/playing up is not included in the text, these would add value to findings and discussion sections.

- Duggan JD, Moody J, Byrne P, McGahan JH, Kirszenstein L. Considerations and guidelines on athletic development for youth Gaelic athletic association players. Strength Cond J. 2022;44(2):76-96.
- Moran, D., Taylor, J., Behan, S., Horgan, P., & MacNamara, Á. (2025). Grouping by Ability in Youth Sport: Streaming in Gaelic Games. Quest, 1-17.

Thank you for highlighting this. We have considered the suggested research and incorporated it into the manuscript.

Please ensure the document is justified.

Thank you for highlighting this. We hope the edits to the manuscript and addition of the suggested research has addressed this concern.

**Specific feedback:**

**Abstract**

Page 2, line 22: level of participant (club, county)

Level of participation has been added to the abstract.

**Updated manuscript:** Semi-structured interviews were conducted with adolescent club Camogie players currently playing up an age grade (n=15; 14.4 ± 1.1 years).

**Introduction**

Page 3, lines 44-45: Doesn’t read well, needs to be re-written

The description of the levels of youth Camogie participation has been updated for clarity.

**Updated manuscript:** Competitive youth Camogie participation typically takes place within formal structures from under-14 onwards (including under-15, under-16, under-17 and/or under-18 age grades). However, many players begin engaging with Camogie at much younger ages (e.g., under-6 or under-8), where early participation focuses on skill development, game-based learning, meaningful playing time and enjoyment of the sport (3).

Page 3, lines 50-51: Is there a percentage drop-out rate specific to Camogie? Would add value here.

Specific details of the dropout rate in youth Camogie have been added to the Introduction.

**Updated manuscript:** Despite the well-documented benefits of participation, over 70% of youth sports participants in Ireland drop out of sport, with 15% dropping out of Camogie specifically, positioning it among the most commonly dropped sports young girls cease participating in (2).

Page 6, line 59-61: Needs a supporting reference

Supporting reference has been added.

**Updated manuscript:** Playing alongside players with a greater chronological, biological and technical age can be beneficial for youth players as more appropriate levels of challenge can lead to better performances and more skilled players (8).

Page 6, line 63-65: Place reference at the end of the sentence

The position of the reference in the sentence has been updated.

**Updated manuscript:** Additionally, there are psychological and social benefits of playing up, such as making new friends and developing social interaction skills (9), which can support adolescents’ social functioning, group cohesion and sport continuation behaviour (4,11).

Page 7, line 63-65: Why is this important during adolescence?

Justification for why this is important during adolescence has been added.

**Updated manuscript:** Additionally, there are psychological and social benefits of playing up, such as making new friends and developing social interaction skills (9), which can support adolescents’ social functioning, group cohesion and sport continuation behaviour (4,11).

**Methodology:**

I’m not a qualitative expert but you will need to provide rationale on why you selected a qualitative phenomenological approach and the semi-structured interviews.

The rationale for the use of a qualitative phenomenological approach and semi-structured interviews has been provided.

**Updated manuscript:** Phenomenology seeks to understand the essence of lived experiences by exploring individuals’ subjective perspectives, enabling a rich and in-depth understanding of what matters to participants and how they make sense of their experiences (13). Qualitative research in this sense specifically aims to integrate the athlete voice and perspective directly into the research (14). Semi-structured interviews allow participants to speak freely about their perceptions and experiences and are led by what is perceived most important to the interviewee (15).

Page 7 Procedures

How were the questions designed on extrapolating the most useful information from the participants?

Further detail has been added to the manuscript for clarity.

**Updated manuscript:** The interview guide was developed by adapting a similar guide utilised in previous research (6) and in line with the 5-step framework for the development of an interview guide (15), ensuring alignment with the research aims, identification of key topics, formulation of open-ended questions and sequencing for logical flow.

**Updated manuscript:** A semi-structured format was employed to allow flexibility, with core questions addressing the central research objectives. In addition, a series of probing questions were incorporated to elicit further clarification and depth, enabling participants to expand on their experiences and ensuring the collection of rich, detailed data directly relevant to the study aims.

**Results**

The overall results section is very long (13 pages), could some of the results be displayed in a table format with specific themes. For example, theme, sub theme and raw data in a table format.

Thank you for this suggestion. We have considerably edited the Results section and added Table 2 to support concise reporting.

Any in-text quotes should be indented, separated and the line spacing should be one. For example:

“maybe if I was a bit bigger, it might help. Maybe if I was just bigger and faster, it would help so maybe starting gym work earlier could be good” (P14).

With the significant edits to the Results section, all long quotes have been removed.

**Discussion**

The discussion section could be drastically improved by incorporating the research in sport on self-determination theory (SDT) and the impact this can have on intrinsic motivation which your results have suggested.

Please see the following references:

- Mallett C. Self determination theory: a case study of evidence-based coaching. Sport Psychol 2005; 19: 417–429.
- Balaguer I, González L, Fabra P, et al. Coaches’ interpersonal style, basic psychological needs and the well- and illbeing of young soccer players: a longitudinal analysis. J Sport Sci 2012; 30: 1619–1629.
- Gillet N, Vallerand RJ, Amoura S, et al. Inﬂuence of coaches’ autonomy support on athletes’ motivation and sport performance: a test of the hierarchical model of intrinsic and extrinsic motivation. Psychol Sport Exerc 2010; 11: 155–161.
- Adie JW, Duba J and Ntoumanis N. Perceived coach-autonomy support, basic need satisfaction and the well- and ill-being of elite youth soccer players: a longitudinal investigation. Psychol Sport Exerc 2012; 13: 1–59.

Thank you for this valued feedback. The Self-Determination Theory (SDT) has been considered throughout the Discussion.

**Updated manuscript:** When interpreted through the lens of the Self-Determination Theory (20,21), the findings highlight the influence of the playing environment on youth players’ intrinsic motivation to play up and participate in Camogie. Although initial challenges were reported, 80% of participants expressed overwhelmingly favourable perceptions of playing up. Most notably, benefits were in relation to their Camogie skill development and psychosocial competence, outcomes that strongly satisfy their need for *competence* as a Camogie player. Despite these benefits, youth Camogie players perceive associated pressures and demands of playing up, adverse peer interactions and impaired intrinsic motivation as negatives of playing up, which actively hinder their need for *autonomy* and *relatedness*.

**Updated manuscript:** This mastery and perceived skill development can directly impact a player's confidence and enjoyment, reinforcing their motivation (24) and commitment to the sport (25). Research in Gaelic games has shown that emphasising the development of key bio-motor qualities (e.g., strength, speed, power), while also creating developmentally appropriate and autonomy-supportive coaching environments can facilitate holistic player development (26).

**Updated manuscript:** In addition, an autonomy-supportive coaching environment that promotes player autonomy, competence and relatedness should be fostered to positively influence the sport experience, improve performance and develop long-term satisfaction in all three Self-Determination Theory basic needs (29,33).

**Updated manuscript:** This also emphasises the importance of autonomy-supportive coaching environments in Gaelic games (26) to ensure athletes’ psychological needs for autonomy (choice), competence (skill development), and relatedness (social connection) are effectively met (39).

**Updated manuscript:** This a breakdown of the relatedness and social fabric of the team, which is a key predictor of positive youth sport experiences and continued participation (55,56).

**Updated manuscript:** Working with youth Camogie players requires awareness of the developmental and psychosocial factors influencing their sporting experiences, with coaches playing a key role in fostering environments that support autonomy, competence and relatedness.

Page 21, line 453-454: what are the significant drop-out rates?

The sentence has been updated to specifically outline the dropout rates.

**Updated manuscript:** Given that almost one in six youth Camogie players dropout of the sport (2), the common practice of age-grade progression, or playing up, warrants examination.

Page 21, 458-461: can these overwhelming positive experiences be reported as percentage values. This really add context to your findings.

We appreciate the reviewer’s suggestion to present these findings as percentage values and have made the necessary changes to this sentence in the Discussion. To note however, we have deliberately avoided quantifying the qualitative data in this manner throughout the manuscript, in line with established methodological guidance (Christou, 2025; Braun & Clarke, 2024; Sandelowski, 2001). Quantification as percentages may oversimplify qualitative data and imply importance based on frequency rather than meaning. Instead, we prioritised depth, context and interpretive richness through thematic analysis and illustrative exemplary quotes.

**Updated manuscript:** Although initial challenges were reported, 80% of participants expressed overwhelmingly favourable perceptions of playing up.

Page 22, 470-471: enhanced learning environment

This sentence has been updated in line with your suggestion.

**Updated manuscript:** The enhanced learning environment can allow players to be consistently pushed beyond their comfort zones when playing up, leading to rapid skill acquisition and tactical understanding (22).

Page 22, 474-476: You have mentioned the physical components important to the game, the following paper makes reference to this and the importance of an autonomous learning environment in developing competent female GAA players. See paragraph on Coaching in this paper.

- Duggan, J. D., Moody, J., Byrne, P., McGahan, J. H., & Kirszenstein, L. (2022). Considerations and guidelines on athletic development for youth Gaelic athletic association players. Strength & Conditioning Journal, 44(2), 76-96.

The manuscript has been updated to incorporate the research by Duggan et al. (2022).

**Updated manuscript:** Research in Gaelic games has shown that emphasising the development of key bio-motor qualities (e.g., strength, speed, power), while also creating developmentally appropriate and autonomy-supportive coaching environments can facilitate holistic player development (26).

**Updated manuscript:** In particular, the development of athletic motor skill competencies (27) through Gaelic games participation can support the development of perceptual and decision-making skills applicable to other sports (26).

**Updated manuscript:** This also emphasises the importance of autonomy-supportive coaching environments in Gaelic games (26) to ensure athletes’ psychological needs for autonomy (choice), competence (skill development), and relatedness (social connection) are effectively met (39).

**Updated manuscript:** Policies should instead incorporate individualised considerations to account for highly variable individual maturation rates and the diverse physical, psychological and developmental needs of young players (26,47,48).

Page 23, 479-485: The aforementioned reference could also support your writing here.

The research by Duggan et al. (2022) has also been considered here.

**Updated manuscript:** In particular, the development of athletic motor skill competencies (27) through Gaelic games participation can support the development of perceptual and decision-making skills applicable to other sports (26).

Page 23, 487-498: A well written paragraph, could be enhanced to linking to the coaching science research on motivation, etc as mentioned previously. The following paper may be of assistance in this area:

- Smith, K., Burns, C., O’Neill, C., Duggan, J. D., Winkelman, N., Wilkie, M., & Coughlan, E. K. (2023). How to coach: A review of theoretical approaches for the development of a novel coach education framework. International Journal of Sports Science & Coaching, 18(2), 594-608.

The paragraph has been updated to link to the available motivation research.

**Updated manuscript:** Performance improvements are facilitated by positive and enjoyable practice activities (28), while a well-designed learning environment that appropriately challenges skill and game development can enhance group connectedness through stronger relationships with coaches and teammates (29).

**Updated manuscript:** In addition, an autonomy-supportive coaching environment that promotes player autonomy, competence and relatedness should be fostered to positively influence the sport experience, improve performance and develop long-term satisfaction in all three Self-Determination Theory basic needs (29,33).

**Updated manuscript:** Consequently, coach education should shift from rigid, prescriptive frameworks regarding *what* to coach toward a facilitative approach focused on *how* to coach (29), while also providing education on training load management, burnout and the psychosocial aspects of youth Camogie participation.

Additionally, practical recommendations could be pulled from this to ensure the GAA are upskilling/educating coaches on the practices in coaching science.

The Practical Implications section has been updated considering the research by Smith et al. (2023).

**Updated manuscript:** Firstly, it highlights the importance of creating challenging environments that motivate young athletes to grow. For example, coaches could utilise small-sided games with specific technical and tactical constraints, implement positional rotation within high-decision-making central zones (midfield, half forward/back), and prioritise process over outcome-oriented goals. While safeguarding against burnout and injury is paramount, providing opportunities for players to test themselves against higher standards can be beneficial. This environment must be underpinned by autonomy-supportive coaching practices. Consequently, coach education should shift from rigid, prescriptive frameworks regarding *what* to coach toward a facilitative approach focused on *how* to coach (29), while also providing education on training load management, burnout and the psychosocial aspects of youth Camogie participation.

Page 24, 513-516: Very long sentence, more concise sentence structure is needed here.

This sentence has been split to improve clarity and readability.

**Updated manuscript:** However, despite evident benefits, for many youth Camogie players, playing up was associated with significant pressures and demands from their coaches and teammates. This often led players to feel overwhelmed by the heightened expectations of the older age group.

Page 25, 536-546: This needs to be support by the research on paediatric physiology and youth athletic development mentioned previously. (Bio-banding, growth and maturation measures, LTAD). Also see the following on the area:

- Ryan, D., Jeffreys, I., Fitzgerl and, F., Moyna, N., O'Connell, A., Murphy, S., ... & Lloyd, R. (2022). Long-term athletic development of Gaelic games players: An action statement. Professional Strength & Conditioning.
- Gaelic Games Player Pathway & Sports Science, 2030 Vision document.

The research on bio-banding and youth athletic development has been considered and added to the manuscript.

**Updated manuscript:** There is a clear need for coaches, parents and organisational leadership to recognise that a uniform approach to playing up an age grade is insufficient. Policies should instead incorporate individualised considerations to account for highly variable individual maturation rates and the diverse physical, psychological and developmental needs of young players (26,47,48). This player-centred ethos aligns directly with the Gaelic Games 2030 Vision (49), which mandates a scientifically informed approach to maximising player potential and retention. Bio-banding, a strategy that groups athletes based on biological maturation rather than chronological age (50), should be considered. However, bio-banding must be grounded in objective measurements, as solely relying on the coaches’ subjective perspective is an invalid method for estimating biological maturation (51). It must also be flexible, as rigid grouping risks excluding late-maturing talent (52). Evidence supports the benefits of such approaches, with youth soccer players reporting that maturity-matched environments are both developmentally and psychologically rewarding (53). Further research specific to Gaelic games is required.

**Practical implications**

Could the GAA as an organization be challenged here to do more? There is plenty of sabre rattling with the 2030 vision document from a coaching science and athletic development perspective but very little has been implemented on the ground. Coaches are often willing to learn and want to support their players but are given little education in the area.

We completely agree with your well-made point here. We have updated this section to include how coach education must be transformed, moving away from ‘traditional’ coaching practices (as outlined by Smith et al., 2023) towards a more diverse pedagogical approach.

**Updated manuscript:** Firstly, it highlights the importance of creating challenging environments that motivate young athletes to grow. For example, coaches could utilise small-sided games with specific technical and tactical constraints, implement positional rotation within high-decision-making central zones (midfield, half forward/back), and prioritise process over outcome-oriented goals. While safeguarding against burnout and injury is paramount, providing opportunities for players to test themselves against higher standards can be beneficial. This environment must be underpinned by autonomy-supportive coaching practices. Consequently, coach education should shift from rigid, prescriptive frameworks regarding *what* to coach toward a facilitative approach focused on *how* to coach (29), while also providing education on training load management, burnout and the psychosocial aspects of youth Camogie participation.

As you have alluded to more education is needed on training load management, burnout, psycho-social aspects and the impact of youth Camogie players playing up.

This has been explicitly stated in the Practical Implications section.

**Updated manuscript:** Consequently, coach education should shift from rigid, prescriptive frameworks regarding *what* to coach toward a facilitative approach focused on *how* to coach (29), while also providing education on training load management, burnout and the psychosocial aspects of youth Camogie participation.

**Reviewer #2**

**Introduction**

The sport of Camogie is introduced well, and you provide a good explanation of age-grades and structure. The introduction progresses with a good flow between topics and good use of positive and negative perspectives.

Thank you for your comments.

**Comment 1 –** I think this section could benefit from emphasising the importance of examining this issue (playing up) specifically in female sport. If there is a lack of literature, please highlight this point.

The prevalence of playing up in youth Camogie has been more explicitly outlined, while also demonstrating that there is a lack of current available evidence in this area.

**Updated manuscript:** Playing up an age grade, considered playing a chronological age grade above the current primary age grade for that player, is common for youth Camogie players, with 52% of under-14 female Gaelic games players playing up to under-16 and 72% of under-16s playing up to under-18 (5).

**Updated manuscript:** Playing up is considerably more prevalent for young females in rural settings in Ireland, with 74% of under-14 players doing so, compared to 22.7% in towns and just 3.3% in cities (5). Despite its prevalence, there is a current lack of research exploring the phenomenon, limiting the extent to which its potential benefits or associated developmental demands can be adequately evaluated.

**Comment 2 –** L44 – “Competitive youth Camogie participation begins at under-14……..” I think this sentence could be a bit misleading. While formal competitive structures (e.g., knockout competitions) may begin at this age grade, many girls begin participating in Camogie at a much earlier stage (e.g., under-8 level). Given that Camogie is a highly skilled sport, early exposure to skill development and games are very important and usually take place. This should be reflected here and avoid the reader thinking that games only start at U14.

Thank you for this comment. This sentence has been updated accordingly to provide clarity on youth Camogie participation levels.

**Updated manuscript:** Competitive youth Camogie participation typically takes place within formal structures from under-14 onwards (including under-15, under-16, under-17 and/or under-18 age grades). However, many players begin engaging with Camogie at much younger ages (e.g., under-6 or under-8), where early participation focuses on skill development, game-based learning, meaningful playing time and enjoyment of the sport (3).

**Comment 3 –** L61-63 – you make reference to the benefits of boy’s youth soccer players, is there any female literature that shows the positive or negatives of playing up? Or what about a ball sport? Is it the same, given that there may be more or less physicality in the game

The literature in this area is currently extremely limited. This study by Kelly et al. (2021) outlined the benefits of playing up for boy’s youth soccer players, to which we have added further research by Williams and MacNamara (2022) in rugby. We did however follow up this point with another available study by Goldman et al. (2022) that explored the benefits of playing up in soccer for both boys and girls. Given the limited available research, it is difficult to make inferences about benefits or negatives based on differences in the physicality of the game. Further research is required to explore this.

**Updated manuscript:** The technical, tactical and/or physical benefits associated with playing up have been demonstrated to date in boys youth soccer (9) and rugby (10). Additionally, there are psychological and social benefits of playing up, such as making new friends and developing social interaction skills (9), which can support adolescents’ social functioning, group cohesion and sport continuation behaviour (4,11). Previous qualitative research in youth boys and girls has identified progress as an athlete as a key benefit of playing up in soccer, with receiving recognition for their talent, experiencing success and having opportunities to develop expertise highlighted (6).

**Comment 4 –** L68-70 – “young athletes”, does this include females? If so, I think it is worth mentioning it. If not, this should be used to highlight the gap in the literature.

Yes, this study included both males and females. This was stated in the previous sentence, but an edit has been made to bring this through to this sentence. Please let us know if further changes are required here to address your comment.

**Updated manuscript:** Previous qualitative research in youth boys and girls has identified progress as an athlete as a key benefit of playing up in soccer, with receiving recognition for their talent, experiencing success and having opportunities to develop expertise highlighted (6). However, these young athletes can find playing up a challenge and struggle most to cope with the intensity of trainings and games, and to fit in socially with older peers (6).

**Comment 5 –** L 76-79 – are you only focusing on the players perspectives? What about informing the coach and management who are selecting the teams?

This study focused solely on the perspective of the youth Camogie player. Our aim was to directly examine the athlete voice and their perceptions of playing up an age grade. The findings of this study, particularly the practical implications, can be used to communicate with the coach and management to inform their practice. However, this has now been added to the limitations.

**Updated manuscript:** The study was also limited to the perspectives of youth players; therefore, future research should incorporate the views of coaches and parents to provide a more comprehensive understanding that reflects all relevant stakeholder perspectives.

**Methods**

The participant description provides useful detail on age, playing experience, previous playing-up experience, and involvement in other sports. The pilot testing of the interview guide adds to the strength of the data collection process.

Thank you for your comments.

**Comment 6 –** L100-106 – Please include how many counties/clubs that players were recruited from, and the size of clubs/or number or teams competing at a certain age-grade within the club. This is important as experiences of playing up may differ depending on club size, county structures, and availability of players.

We agree this would be beneficial information to include but at the conclusion of the project, as per ethical approval, the transcripts were completely anonymised, and it is therefore not possible to now add this information to the manuscript. However, a key feature of our recruitment strategy was to ensure diversity of participants. With this in mind, we ensured there was a balance of counties and provinces represented within the sample, as well as diverse representation of urban and rural clubs. This has been added to the manuscript.

**Updated manuscript:** A key element of the recruitment strategy was to ensure diversity of participants from urban and rural clubs across counties and provinces.

**Comment 7 –** L109 – within the procedures, clearly state the exact inclusion criteria for playing up. For example, did all participants play one year up, two years up, or multiple grades? Was this at club, county, school, or all contexts?

The inclusion criteria has been updated in the Procedures section.

**Updated manuscript:** Inclusion criteria specified that participants were required to be a youth Camogie player currently competing at an adolescent age grade at club level. While there was no requirement for participants to be playing up an age grade (in order to capture perspectives from both those who do and do not engage in this practice), notably all participants in the final sample were currently playing up an age grade.

**Comment 8 –** Please include the average duration and range of the interviews. This would provide useful detail on the depth of data collected from participants.

We agree this would be beneficial information to include but as per comment 6, at the conclusion of the project, the transcripts were completely anonymised and the audio recordings were disposed of, and it is therefore not possible to now add this information. However, all interviews were a minimum of 40 minutes in duration, and this has been added to the Procedures section.

**Updated manuscript:** All interviews were a minimum of 40 minutes in duration.

**Comment 9 –** Were all interviews included? All questions answered? If not, how did you deal with them?

This has been updated in the manuscript.

**Updated manuscript:** All questions were answered by participants, and all interviews were included for analysis.

**Comment 10 –** L125 – pilot interviews were conducted with female Gaelic football players rather than Camogie players. What was the reason for this? While acceptable, it should be justified because the topic is specific to Camogie. Please provide a short rationale for this choice and explain whether any Camogie-specific language or questions were reviewed before the main interviews.

Further detail on the pilot study has been added.

**Updated manuscript:** This population was chosen for the pilot study due to the similarity in structure, terminology and developmental pathways across youth female Gaelic games, which allowed for effective evaluation of question clarity, relevance and flow in a comparable context. This approach also ensured that the Camogie player sample required for the main study was not compromised during piloting.

**Updated manuscript:** The interview guide was subsequently reviewed and adapted to ensure that all language, terminology and examples were specific to Camogie before the main interviews were conducted.

**Results**

The Results section provides valuable insight into the players’ experiences of playing up. The addition of players quotes provides the athlete’s voice. However, the section seems quite lengthy and descriptive in places.

Thank you for your comments. We have significantly edited the length of the results section to enhance readability.

**Updated manuscript:** See Results section in manuscript.

**Comment 11 –** I recommend tightening the Results by reducing repetition, especially where similar ideas appear across multiple themes, such as social inclusion, physical challenge, and pressure.

We have considerably edited the Results section with this in mind. Thank you for your suggestions.

**Updated manuscript:** See Results section in manuscript.

**Comment 12 –** The “Recommendations” section is valuable, but some of this material may be better shortened in the Results and expanded in the Practical Implications section.

The recommendations reported in the Results have been edited and expanded upon in the Practical Implications section.

**Updated manuscript:** See Practical Implications section in manuscript.

**Discussion**

The Discussion is well structured and effectively links the findings to existing literature. However, in places it remains descriptive and repeats elements of the Results section.

The Discussion has been edited, with the significant addition of relevant available literature.

**Updated manuscript:** See Discussion section in manuscript.

**Comment 13 –** I would encourage the authors to place greater emphasis on interpretation, focusing on why these findings occur and what they mean for youth athlete development, especially females.

Thank you for this comment. The Discussion has been updated, and we believe we have clarified the interpretation of the findings. Please direct us if you believe further clarity and interpretation is required.

**Updated manuscript:** See Discussion section in manuscript.

**Practical Applications**

The practical implications are relevant and clearly linked to youth player development. However, they the statements are general in places.

Thank you for your feedback. We have updated the Practical Implications section to provide more explicit recommendations rather than simply providing broad statements.

**Updated manuscript:** See Practical Implications section in manuscript.

**Comment 14 –** I would encourage the authors to provide more specific, actionable recommendations for coaches like what was suggested including team-building activities.

Thank you for this suggestion. We have updated the Practical Implications with more actionable recommendations.

**Updated manuscript:** See Practical Implications section in manuscript.

Others for example:

L581 – how can coaches create challenging environments?

Specific examples of how coaches can create challenging environments has been added.

**Updated manuscript:** Firstly, it highlights the importance of creating challenging environments that motivate young athletes to grow. For example, coaches could utilise small-sided games with specific technical and tactical constraints, implement positional rotation within high-decision-making central zones (midfield, half forward/back), and prioritise process over outcome-oriented goals.

L582 – how can coaches help avoid burnout?

Further detail on coach education has been added.

**Updated manuscript:** Consequently, coach education should shift from rigid, prescriptive frameworks regarding *what* to coach toward a facilitative approach focused on *how* to coach (29), while also providing education on training load management, burnout and the psychosocial aspects of youth Camogie participation.

L585 – how would coaches know that players about “individual readiness and psychological maturity?” The addition of these suggestions would improve its usability for practitioners.

Practical suggestions have been added.

**Updated manuscript:** Secondly, coaches should consider individual readiness and psychological maturity, not just chronological age or number shortages, when making decisions about player participation. Identifying players who thrive in more challenging settings and offering them appropriate opportunities could optimise their development. Coaches can assess readiness by evaluating emotional regulation, self-efficacy and social adaptability through structured trial exposures, collaborative feedback and regular player check-ins. Crucially, these practices must be underpinned by an autonomy-supportive and psychologically safe environment that prioritises player learning without fear of making mistakes.

**Limitations**

**Comment 15 –** A key limitation is the sample size and the personal perspectives of those sampled have for playing up. In addition, the study only focused on players and did not collect perspectives from coaches and parents, which limits the broader understanding of the topic. Please add these to the section.

Thank you for this comment. We have now included how the study focused solely on the personal perspective of players. However, we do not consider the sample size to be a limitation, as qualitative research commonly supports smaller samples for in-depth inquiry, with studies suggesting that approximately 6-20 participants are sufficient for semi-structured interviews (e.g., Guest et al., 2020; Braun & Clarke, 2013). Furthermore, the sample size was guided by the concept of information power (Malterud et al., 2016), whereby the adequacy of the sample is determined by the richness, relevance and specificity of the data in relation to the study aims, rather than by numerical thresholds alone.

**Updated manuscript:** This study solely considered the personal perspective of players currently playing up but not those who wish to play up but are not selected, or those adversely affected by younger teammates playing up.

**Updated manuscript:** The study was also limited to the perspectives of youth players; therefore, future research should incorporate the views of coaches and parents to provide a more comprehensive understanding that reflects all relevant stakeholder perspectives.
