# Supplementary material for: Stepping up: A qualitative examination of youth Camogie players’ perceptions of playing up an age grade
Source: PLoS One. 2026 Aug 11;21(8):e0355563. doi: 10.1371/journal.pone.0355563 (PMC13460593; doi:10.1371/journal.pone.0355563)
Supplement: Supplementary file 4 [file pone.0355563.s002.docx]

Review: **Stepping Up: A Qualitative Examination of Youth Camogie Players’ Perceptions of Playing Up an Age Grade**

I would like to thank the editorial board for the opportunity to review the current manuscript titled “*Stepping Up: A Qualitative Examination of Youth Camogie Players’ Perceptions of Playing Up an Age Grade”.*

The study is well structured manuscript that explored its stated aim through the use of semi-structured interviews, capturing both the perceived benefits and challenges associated with playing up in Camogie. The inclusion of player quotations is valuable. Overall, this is a novel and practically relevant study with clear implications for coaches, parents, and the Camogie Association who organise the game. With some minor adjustments, the manuscript has the potential to make a meaningful contribution to both research and applied practice in Camogie.

**Overall**

I have outlined a number of comments below which I hope will assist in refining the manuscript.

The following are comments that need to be addressed:

**Introduction**

The sport of Camogie is introduced well, and you provide a good explanation of age-grades and structure. The introduction progresses with a good flow between topics and good use of positive and negative perspectives.

Comment 1 - I think this section could benefit from emphasising the importance of examining this issue (playing up) specifically in female sport. If there is a lack of literature, please highlight this point.

Comment 2 – L44 – “Competitive youth Camogie participation begins at under-14……..” I think this sentence could be a bit misleading. While formal competitive structures (e.g., knockout competitions) may begin at this age grade, many girls begin participating in Camogie at a much earlier stage (e.g., under-8 level). Given that Camogie is a highly skilled sport, early exposure to skill development and games are very important and usually take place. This should be reflected here and avoid the reader thinking that games only start at U14.

Comment 3 – L61-63 – you make reference to the benefits of boy’s youth soccer players, is there any female literature that shows the positive or negatives of playing up? Or what about a ball sport? Is it the same, given that there may be more or less physicality in the game

Comment 4 – L68-70 – “young athletes”, does this include females? If so, I think it is worth mentioning it. If not, this should be used to highlight the gap in the literature.

Comment 5 – L 76-79 – are you only focusing on the players perspectives? What about informing the coach and management who are selecting the teams?

**Methods**

The participant description provides useful detail on age, playing experience, previous playing-up experience, and involvement in other sports.

The pilot testing of the interview guide adds to the strength of the data collection process.

Comment 6 – L100-106 – Please include how many counties/clubs that players were recruited from, and the size of clubs/or number or teams competing at a certain age-grade within the club. This is important as experiences of playing up may differ depending on club size, county structures, and availability of players.

Comment 7 – L109 – within the procedures, clearly state the exact inclusion criteria for playing up. For example, did all participants play one year up, two years up, or multiple grades? Was this at club, county, school, or all contexts?

Comment 8 - Please include the average duration and range of the interviews. This would provide useful detail on the depth of data collected from participants.

Comment 9 - Were all interviews included? All questions answered? If not, how did you deal with them?

Comment 10 – L125 – pilot interviews were conducted with female Gaelic football players rather than Camogie players. What was the reason for this? While acceptable, it should be justified because the topic is specific to Camogie. Please provide a short rationale for this choice and explain whether any Camogie-specific language or questions were reviewed before the main interviews.

**Results**

The Results section provides valuable insight into the players’ experiences of playing up. The addition of players quotes provides the athlete’s voice. However, the section seems quite lengthy and descriptive in places.

Comment 11 – I recommend tightening the Results by reducing repetition, especially where similar ideas appear across multiple themes, such as social inclusion, physical challenge, and pressure.

Comment 12 – The “Recommendations” section is valuable, but some of this material may be better shortened in the Results and expanded in the Practical Implications section.

**Discussion**

The Discussion is well structured and effectively links the findings to existing literature. However, in places it remains descriptive and repeats elements of the Results section.

Comment 13 – I would encourage the authors to place greater emphasis on interpretation, focusing on why these findings occur and what they mean for youth athlete development, especially females.

**Practical Applications**

The practical implications are relevant and clearly linked to youth player development. However, they the statements are general in places.

Comment 14 – I would encourage the authors to provide more specific, actionable recommendations for coaches like what was suggested including team-building activities. Others for example:

L581 – how can coaches create challenging environments?

L582 – how can coaches help avoid burnout?

L585 – how would coaches know that players about “individual readiness and psychological maturity?”

The addition of these suggestions would improve its usability for practitioners.

**Limitations**

Comment 15 – A key limitation is the sample size and the personal perspectives of those sampled have for playing up. In addition, the study only focused on players and did not collect perspectives from coaches and parents, which limits the broader understanding of the topic. Please add these to the section
