# Supplementary material for: Stepping up: A qualitative examination of youth Camogie players’ perceptions of playing up an age grade
Source: PLoS One. 2026 Aug 11;21(8):e0355563. doi: 10.1371/journal.pone.0355563 (PMC13460593; doi:10.1371/journal.pone.0355563)
Supplement: Peer review history

# Peer review: Decision Letter 0

**DOI:** 10.1371/journal.pone.0355563.r001

**Authors:** Shane Malone

5 May 2026

PONE-D-26-14972

Stepping Up: A Qualitative Examination of Youth Camogie Players’ Perceptions of Playing Up an Age Grade

PLOS One

Dear Dr. O'Keeffe,

Thank you for submitting your manuscript to PLOS ONE. After careful consideration, we feel that it has merit but does not fully meet PLOS ONE’s publication criteria as it currently stands. Therefore, we invite you to submit a revised version of the manuscript that addresses the points raised during the review process.

Overall, the reviewers feel that the manuscript requires a mixture of minor and major edits. Please complete these as best as possible, and we will endeavour to get this paper into publication with PLOS ONE. The paper is a worthy addition to the Gaelic Sports literature; however, it needs to be tightened up in specific areas.

Please submit your revised manuscript by Jun 19 2026 11:59PM. If you will need more time than this to complete your revisions, please reply to this message or contact the journal office at plosone@plos.org. When you're ready to submit your revision, log on to https://www.editorialmanager.com/pone/ and select the 'Submissions Needing Revision' folder to locate your manuscript file.

Please include the following items when submitting your revised manuscript:

- A letter that responds to each point raised by the academic editor and reviewer(s). You should upload this letter as a separate file labeled 'Response to Reviewers'.
- A marked-up copy of your manuscript that highlights changes made to the original version. You should upload this as a separate file labeled 'Revised Manuscript with Track Changes'.
- An unmarked version of your revised paper without tracked changes. You should upload this as a separate file labeled 'Manuscript'.

If you would like to make changes to your financial disclosure, please include your updated statement in your cover letter. Guidelines for resubmitting your figure files are available below the reviewer comments at the end of this letter.

If applicable, we recommend that you deposit your laboratory protocols in protocols.io to enhance the reproducibility of your results. Protocols.io assigns your protocol its own identifier (DOI) so that it can be cited independently in the future. For instructions see: https://journals.plos.org/plosone/s/submission-guidelines#loc-laboratory-protocols. Additionally, PLOS ONE offers an option for publishing peer-reviewed Lab Protocol articles, which describe protocols hosted on protocols.io. Read more information on sharing protocols at https://plos.org/protocols?utm_medium=editorial-email&utm_source=authorletters&utm_campaign=protocols.

As the corresponding author, your ORCID iD is verified in the submission system and will appear in the published article. PLOS supports the use of ORCID, and we encourage all coauthors to register for an ORCID iD and use it as well. Please encourage your coauthors to verify their ORCID iD within the submission system before final acceptance, as unverified ORCID iDs will not appear in the published article. *Only*  the individual author can complete the verification step; PLOS staff *cannot*  verify ORCID iDs on behalf of authors.

We look forward to receiving your revised manuscript.

Kind regards,

Shane Malone, Ph.D.

Academic Editor

PLOS One

Journal Requirements:

When submitting your revision, we need you to address these additional requirements.

1. Please ensure that your manuscript meets PLOS ONE's style requirements, including those for file naming. The PLOS ONE style templates can be found at

https://journals.plos.org/plosone/s/file?id=wjVg/PLOSOne_formatting_sample_main_body.pdf and

https://journals.plos.org/plosone/s/file?id=ba62/PLOSOne_formatting_sample_title_authors_affiliations.pdf.

2. Please provide additional details regarding participant consent. In the ethics statement in the Methods and online submission information, please ensure that you have specified (1) whether consent was informed and (2) what type you obtained (for instance, written or verbal, and if verbal, how it was documented and witnessed). If your study included minors, state whether you obtained consent from parents or guardians. If the need for consent was waived by the ethics committee, please include this information.

If you are reporting a retrospective study of medical records or archived samples, please ensure that you have discussed whether all data were fully anonymized before you accessed them and/or whether the IRB or ethics committee waived the requirement for informed consent. If patients provided informed written consent to have data from their medical records used in research, please include this information.

3. Thank you for stating the following financial disclosure:

“This research was funded by Sport Ireland. “

Please state what role the funders took in the study.  If the funders had no role, please state: "The funders had no role in study design, data collection and analysis, decision to publish, or preparation of the manuscript."

If this statement is not correct you must amend it as needed.

Please include this amended Role of Funder statement in your cover letter; we will change the online submission form on your behalf.

4. We note that you have indicated that there are restrictions to data sharing for this study. For studies involving human research participant data or other sensitive data, we encourage authors to share de-identified or anonymized data. However, when data cannot be publicly shared for ethical reasons, we allow authors to make their data sets available upon request. For information on unacceptable data access restrictions, please see http://journals.plos.org/plosone/s/data-availability#loc-unacceptable-data-access-restrictions.

Before we proceed with your manuscript, please address the following prompts:

a) If there are ethical or legal restrictions on sharing a de-identified data set, please explain them in detail (e.g., data contain potentially identifying or sensitive patient information, data are owned by a third-party organization, etc.) and who has imposed them (e.g., a Research Ethics Committee or Institutional Review Board, etc.). Please also provide contact information for a data access committee, ethics committee, or other institutional body to which data requests may be sent.

b) If there are no restrictions, please upload the minimal anonymized data set necessary to replicate your study findings to a stable, public repository and provide us with the relevant URLs, DOIs, or accession numbers. Please see http://www.bmj.com/content/340/bmj.c181.long for guidelines on how to de-identify and prepare clinical data for publication. For a list of recommended repositories, please see https://journals.plos.org/plosone/s/recommended-repositories. You also have the option of uploading the data as Supporting Information files, but we would recommend depositing data directly to a data repository if possible.

Please update your Data Availability statement in the submission form accordingly.

5. Please be informed that funding information should not appear in the Acknowledgments section or other areas of your manuscript. We will only publish funding information present in the Funding Statement section of the online submission form. Please remove any funding-related text from the manuscript.

6. Please include captions for your Supporting Information files at the end of your manuscript, and update any in-text citations to match accordingly. Please see our Supporting Information guidelines for more information: http://journals.plos.org/plosone/s/supporting-information.

7. If the reviewer comments include a recommendation to cite specific previously published works, please review and evaluate these publications to determine whether they are relevant and should be cited. There is no requirement to cite these works unless the editor has indicated otherwise.

[Note: HTML markup is below. Please do not edit.]

Reviewers' comments:

Reviewer's Responses to Questions

**Comments to the Author**

1. Is the manuscript technically sound, and do the data support the conclusions?

The manuscript must describe a technically sound piece of scientific research with data that supports the conclusions. Experiments must have been conducted rigorously, with appropriate controls, replication, and sample sizes. The conclusions must be drawn appropriately based on the data presented.

Reviewer #1: Partly

Reviewer #2: Yes

**********

2. Has the statistical analysis been performed appropriately and rigorously?

Reviewer #1: N/A

Reviewer #2: Yes

**********

3. Have the authors made all data underlying the findings in their manuscript fully available?

The PLOS Data policy requires authors to make all data underlying the findings described in their manuscript fully available without restriction, with rare exception (please refer to the Data Availability Statement in the manuscript PDF file). The data should be provided as part of the manuscript or its supporting information, or deposited to a public repository. For example, in addition to summary statistics, the data points behind means, medians and variance measures should be available. If there are restrictions on publicly sharing data—e.g. participant privacy or use of data from a third party—those must be specified.

Reviewer #1: Yes

Reviewer #2: Yes

**********

4. Is the manuscript presented in an intelligible fashion and written in standard English?

PLOS ONE does not copyedit accepted manuscripts, so the language in submitted articles must be clear, correct, and unambiguous. Any typographical or grammatical errors should be corrected at revision, so please note any specific errors here.

Reviewer #1: Yes

Reviewer #2: Yes

**********

5. Review Comments to the Author

Please use the space provided to explain your answers to the questions above. You may also include additional comments for the author, including concerns about dual publication, research ethics, or publication ethics. (Please upload your review as an attachment if it exceeds 20,000 characters)

Reviewer #1: General feedback:

Thank you for the opportunity to review this paper. This area of underage Camogie is under researched, and this information could potentially add to the field. This is a very well written piece of work and the authors should be commended for their input into the paper.

There is still some work required to ensure you are maximizing the informative results you have extrapolated from the data. There is an opportunity here for you to provided recommendations to the GAA and coaches from the best practices from coaching science and sports psychology. This could be an important practical application of your research for coaches/practitioners to prepare youth Camogie players to stream & play up.

The results section is far too long, and I’ve provided recommendations and feedback in the comments below.

The results section is far too long, please specific feedback below on streamlining this section.

The authors should refine their discussion section before the manuscript can be reconsidered. The discussion section makes broad recommendations, this needs to be specific to your findings with support from the sports science and coaching science literature (See discussion section below).

The authors may consider incorporating the concept of bio-banding, physical development into the discussion section using the following research papers:

Fitzgerald, F., Campbell, M., Kearney, P. E., & Cumming, S. P. (2025). The eyes don’t have it: Coaches’ eye is not a valid method of estimating biological maturation in male Gaelic football. International Journal of Sports Science & Coaching, 20(1), 35-44.

Kearney, C., Coughlan, E. K., O'Connell, A., Lacey, S., & Burns, C. (2025). Bigger but are they better? Investigating growth, maturation, and physical performance metrics in underage Gaelic games development squads. The Journal of Strength & Conditioning Research, 39(6), e806-e814.

Under-researched area in GAA and especially in Camogie.

Prof Sean Cumming also has some research on players opinions on playing up in the academy football could be included in the discussion:

Bradley, B., Johnson, D., Hill, M., McGee, D., Kana-Ah, A., Sharpin, C., ... & Malina, R. M. (2019). Bio-banding in academy football: player’s perceptions of a maturity matched tournament. Annals of human biology, 46(5), 400-408.

There are some minor formatting issues throughout the document, please ensure they are addressed before re-submission.

Concerningly, some of the seminal research in GAA, streaming/playing up is not included in the text, these would add value to findings and discussion sections.

Duggan JD, Moody J, Byrne P, McGahan JH, Kirszenstein L. Considerations and guidelines on athletic development for youth Gaelic athletic association players. Strength Cond J. 2022;44(2):76-96.

Moran, D., Taylor, J., Behan, S., Horgan, P., & MacNamara, Á. (2025). Grouping by Ability in Youth Sport: Streaming in Gaelic Games. Quest, 1-17.

Please ensure the document is justified.

Specific feedback:

Abstract

Page 2, line 22: level of participant (club, county)

Introduction

Page 3, lines 44-45: Doesn’t read well, needs to be re-written

Page 3, lines 50-51: Is there a percentage drop-out rate specific to Camogie? Would add value here.

Page 6, line 59-61: Needs a supporting reference

Page 6, line 63-65: Place reference at the end of the sentence

Page 7, line 63-65: Why is this important during adolescence?

Methodology:

I’m not a qualitative expert but you will need to provide rationale on why you selected a qualitative phenomenological approach and the semi-structured interviews.

Page 7 Procedures

How were the questions designed on extrapolating the most useful information from the participants.

Results

The overall results section is very long (13 pages), could some of the results be displayed in a table format with specific themes. For example, theme, sub theme and raw data in a table format.

Any in-text quotes should be indented, separated and the line spacing should be one.

For example:

“maybe if I was a bit bigger, it might help. Maybe if I was just bigger and faster, it would help so maybe starting gym work earlier could be good” (P14).

Discussion

The discussion section could be drastically improved by incorporating the research in sport on self-determination theory (SDT) and the impact this can have on intrinsic motivation which your results have suggest.

Please see the following references:

Mallett C. Self determination theory: a case study of evidence-based coaching. Sport Psychol 2005; 19: 417–429.

Balaguer I, González L, Fabra P, et al. Coaches’ interpersonal style, basic psychological needs and the well- and illbeing of young soccer players: a longitudinal analysis. J Sport Sci 2012; 30: 1619–1629.

Gillet N, Vallerand RJ, Amoura S, et al. In�uence of coaches’ autonomy support on athletes’ motivation and sport performance: a test of the hierarchical model of intrinsic and extrinsic motivation. Psychol Sport Exerc 2010; 11: 155–161.

*Adie JW, Duba J and Ntoumanis N. Perceived coach-autonomy support, basic need satisfaction and the well- and ill-being of elite youth soccer players: a longitudinal investigation. Psychol Sport Exerc 2012; 13: 1–59.

Page 21, line 453-454: what are the significant drop-out rates?

Page 21, 458-461: can these overwhelming positive experiences be reported as percentage values. This really add context to your findings.

Page 22, 470-471: enhanced learning environment

Page 22, 474-476: You have mentioned the physical components important to the game, the following paper makes reference to this and the importance of an autonomous learning environment in developing competent female GAA players:

Duggan, J. D., Moody, J., Byrne, P., McGahan, J. H., & Kirszenstein, L. (2022). Considerations and guidelines on athletic development for youth Gaelic athletic association players. Strength & Conditioning Journal, 44(2), 76-96.

See paragraph on Coaching in this paper.

Page 23, 479-485: The aforementioned reference could also support your writing here.

Page 23, 487-498: A well written paragraph, could be enhanced to linking to the coaching science research on motivation, etc as mentioned previously:

The following paper maybe of assistance in this area:

Smith, K., Burns, C., O’Neill, C., Duggan, J. D., Winkelman, N., Wilkie, M., & Coughlan, E. K. (2023). How to coach: A review of theoretical approaches for the development of a novel coach education framework. International Journal of Sports Science & Coaching, 18(2), 594-608.

Additionally, practical recommendations could be pulled from this to ensure the GAA are upskilling/educating coaches on the practices in coaching science.

Page 24, 513-516: Very long sentence, more concise sentence structure is needed here.

Page 25, 536-546: This needs to be support by the research on paediatric physiology and youth athletic development mentioned previously. (Bio-banding, growth and maturation measures, LTAD)

Also see the following on the area:

Ryan, D., Jeffreys, I., Fitzgerl and, F., Moyna, N., O'Connell, A., Murphy, S., ... & Lloyd, R. (2022). Long-term athletic development of Gaelic games players: An action statement. Professional Strength & Conditioning.

Game Games Player Pathway & Sports Science, 2030 Vision document.

Practical implications

Could the GAA as an organization be challenged here to do more? There is plenty of sabre rattling with the 2030 vision document from a coaching science and athletic development perspective but very little has been implemented on the ground. Coaches are often willing to learn and want to support their players but are given little education in the area.

As you have alluded to more education is needed on training load management, burnout, psycho-social aspects and the impact of youth Camogie players playing up.

Reviewer #2: Review: Stepping Up: A Qualitative Examination of Youth Camogie Players’ Perceptions of Playing Up an Age Grade

I would like to thank the editorial board for the opportunity to review the current manuscript titled “Stepping Up: A Qualitative Examination of Youth Camogie Players’ Perceptions of Playing Up an Age Grade”.

The study is well structured manuscript that explored its stated aim through the use of semi-structured interviews, capturing both the perceived benefits and challenges associated with playing up in Camogie. The inclusion of player quotations is valuable. Overall, this is a novel and practically relevant study with clear implications for coaches, parents, and the Camogie Association who organise the game. With some minor adjustments, the manuscript has the potential to make a meaningful contribution to both research and applied practice in Camogie.

Overall

I have outlined a number of comments below which I hope will assist in refining the manuscript.

The following are comments that need to be addressed:

Introduction

The sport of Camogie is introduced well, and you provide a good explanation of age-grades and structure. The introduction progresses with a good flow between topics and good use of positive and negative perspectives.

Comment 1 - I think this section could benefit from emphasising the importance of examining this issue (playing up) specifically in female sport. If there is a lack of literature, please highlight this point.

Comment 2 – L44 – “Competitive youth Camogie participation begins at under-14……..” I think this sentence could be a bit misleading. While formal competitive structures (e.g., knockout competitions) may begin at this age grade, many girls begin participating in Camogie at a much earlier stage (e.g., under-8 level). Given that Camogie is a highly skilled sport, early exposure to skill development and games are very important and usually take place. This should be reflected here and avoid the reader thinking that games only start at U14.

Comment 3 – L61-63 – you make reference to the benefits of boy’s youth soccer players, is there any female literature that shows the positive or negatives of playing up? Or what about a ball sport? Is it the same, given that there may be more or less physicality in the game

Comment 4 – L68-70 – “young athletes”, does this include females? If so, I think it is worth mentioning it. If not, this should be used to highlight the gap in the literature.

Comment 5 – L 76-79 – are you only focusing on the players perspectives? What about informing the coach and management who are selecting the teams?

Methods

The participant description provides useful detail on age, playing experience, previous playing-up experience, and involvement in other sports.

The pilot testing of the interview guide adds to the strength of the data collection process.

Comment 6 – L100-106 – Please include how many counties/clubs that players were recruited from, and the size of clubs/or number or teams competing at a certain age-grade within the club. This is important as experiences of playing up may differ depending on club size, county structures, and availability of players.

Comment 7 – L109 – within the procedures, clearly state the exact inclusion criteria for playing up. For example, did all participants play one year up, two years up, or multiple grades? Was this at club, county, school, or all contexts?

Comment 8 - Please include the average duration and range of the interviews. This would provide useful detail on the depth of data collected from participants.

Comment 9 - Were all interviews included? All questions answered? If not, how did you deal with them?

Comment 10 – L125 – pilot interviews were conducted with female Gaelic football players rather than Camogie players. What was the reason for this? While acceptable, it should be justified because the topic is specific to Camogie. Please provide a short rationale for this choice and explain whether any Camogie-specific language or questions were reviewed before the main interviews.

Results

The Results section provides valuable insight into the players’ experiences of playing up. The addition of players quotes provides the athlete’s voice. However, the section seems quite lengthy and descriptive in places.

Comment 11 – I recommend tightening the Results by reducing repetition, especially where similar ideas appear across multiple themes, such as social inclusion, physical challenge, and pressure.

Comment 12 – The “Recommendations” section is valuable, but some of this material may be better shortened in the Results and expanded in the Practical Implications section.

Discussion

The Discussion is well structured and effectively links the findings to existing literature. However, in places it remains descriptive and repeats elements of the Results section.

Comment 13 – I would encourage the authors to place greater emphasis on interpretation, focusing on why these findings occur and what they mean for youth athlete development, especially females.

Practical Applications

The practical implications are relevant and clearly linked to youth player development. However, they the statements are general in places.

Comment 14 – I would encourage the authors to provide more specific, actionable recommendations for coaches like what was suggested including team-building activities. Others for example:

L581 – how can coaches create challenging environments?

L582 – how can coaches help avoid burnout?

L585 – how would coaches know that players about “individual readiness and psychological maturity?”

The addition of these suggestions would improve its usability for practitioners.

Limitations

Comment 15 – A key limitation is the sample size and the personal perspectives of those sampled have for playing up. In addition, the study only focused on players and did not collect perspectives from coaches and parents, which limits the broader understanding of the topic. Please add these to the section

**********

6. PLOS authors have the option to publish the peer review history of their article (what does this mean?). If published, this will include your full peer review and any attached files.

If you choose “no”, your identity will remain anonymous but your review may still be made public.

**Do you want your identity to be public for this peer review?**  For information about this choice, including consent withdrawal, please see our Privacy Policy.

Reviewer #1: **Yes:**  Dr John David Duggan

Reviewer #2: No

**********

[NOTE: If reviewer comments were submitted as an attachment file, they will be attached to this email and accessible via the submission site. Please log into your account, locate the manuscript record, and check for the action link "View Attachments". If this link does not appear, there are no attachment files.]

To ensure your figures meet our technical requirements, please review our figure guidelines: https://journals.plos.org/plosone/s/figures

You may also use PLOS’s free figure tool, NAAS, to help you prepare publication quality figures: https://journals.plos.org/plosone/s/figures#loc-tools-for-figure-preparation.

NAAS will assess whether your figures meet our technical requirements by comparing each figure against our figure specifications.

Attachment Submitted filename: Camogie _Playing Up_Review Comments.docx

# Author comment: Author response to Decision Letter 1

**DOI:** 10.1371/journal.pone.0355563.r002

23 Jun 2026

We would like to thank the reviewers for their time and consideration of our manuscript. The comments were incredibly helpful and insightful. We have considered the comments and specific feedback provided and have made considerable edits. We hope the edits we have made are sufficient in making this manuscript suitable for publication.

Attachment Submitted filename: Response to Reviewers (PONE-D-26-14972).docx

# Peer review: Decision Letter 1

**DOI:** 10.1371/journal.pone.0355563.r003

**Authors:** Shane Malone

23 Jul 2026

Stepping Up: A Qualitative Examination of Youth Camogie Players’ Perceptions of Playing Up an Age Grade

PONE-D-26-14972R1

Dear Dr. O'Keeffe,

We’re pleased to inform you that your manuscript has been judged scientifically suitable for publication and will be formally accepted for publication once it meets all outstanding technical requirements.

Within one week, you’ll receive an e-mail detailing the required amendments. When these have been addressed, you’ll receive a formal acceptance letter and your manuscript will be scheduled for publication.

An invoice will be generated when your article is formally accepted. Please note, if your institution has a publishing partnership with PLOS and your article meets the relevant criteria, all or part of your publication costs will be covered. Please make sure your user information is up-to-date by logging into Editorial Manager at Editorial Manager® and clicking the ‘Update My Information' link at the top of the page. For questions related to billing, please contact billing support.

If your institution or institutions have a press office, please notify them about your upcoming paper to help maximize its impact. If they’ll be preparing press materials, please inform our press team as soon as possible -- no later than 48 hours after receiving the formal acceptance. Your manuscript will remain under strict press embargo until 2 pm Eastern Time on the date of publication. For more information, please contact onepress@plos.org.

Kind regards,

Shane Malone, Ph.D.

Academic Editor

PLOS One

Additional Editor Comments (optional):

Reviewers' comments:

Reviewer's Responses to Questions

**Comments to the Author**

1. If the authors have adequately addressed your comments raised in a previous round of review and you feel that this manuscript is now acceptable for publication, you may indicate that here to bypass the “Comments to the Author” section, enter your conflict of interest statement in the “Confidential to Editor” section, and submit your "Accept" recommendation.

Reviewer #1: All comments have been addressed

Reviewer #2: All comments have been addressed

**********

2. Is the manuscript technically sound, and do the data support the conclusions?

The manuscript must describe a technically sound piece of scientific research with data that supports the conclusions. Experiments must have been conducted rigorously, with appropriate controls, replication, and sample sizes. The conclusions must be drawn appropriately based on the data presented.

Reviewer #1: Yes

Reviewer #2: Yes

**********

3. Has the statistical analysis been performed appropriately and rigorously?

Reviewer #1: Yes

Reviewer #2: Yes

**********

4. Have the authors made all data underlying the findings in their manuscript fully available?

The PLOS Data policy requires authors to make all data underlying the findings described in their manuscript fully available without restriction, with rare exception (please refer to the Data Availability Statement in the manuscript PDF file). The data should be provided as part of the manuscript or its supporting information, or deposited to a public repository. For example, in addition to summary statistics, the data points behind means, medians and variance measures should be available. If there are restrictions on publicly sharing data—e.g. participant privacy or use of data from a third party—those must be specified.

Reviewer #1: Yes

Reviewer #2: Yes

**********

5. Is the manuscript presented in an intelligible fashion and written in standard English?

PLOS ONE does not copyedit accepted manuscripts, so the language in submitted articles must be clear, correct, and unambiguous. Any typographical or grammatical errors should be corrected at revision, so please note any specific errors here.

Reviewer #1: Yes

Reviewer #2: Yes

**********

6. Review Comments to the Author

Please use the space provided to explain your answers to the questions above. You may also include additional comments for the author, including concerns about dual publication, research ethics, or publication ethics. (Please upload your review as an attachment if it exceeds 20,000 characters)

Reviewer #1: Thank you for addressing the comments in the first revision. This will add to the literature available currently in Camogie.

Reviewer #2: Thank you for addressing the suggested comments. You have clearly described your rationale for each

**********

7. PLOS authors have the option to publish the peer review history of their article (what does this mean?). If published, this will include your full peer review and any attached files.

If you choose “no”, your identity will remain anonymous but your review may still be made public.

**Do you want your identity to be public for this peer review?**  For information about this choice, including consent withdrawal, please see our Privacy Policy.

Reviewer #1: No

Reviewer #2: No

**********
